# Exploring lateral genetic transfer among microbial genomes using TF-IDF

**DOI:** 10.1038/srep29319

**Published:** 2016-07-25

**Authors:** Yingnan Cong, Yao-ban Chan, Mark A. Ragan

**Affiliations:** 1 Institute for Molecular Bioscience and ARC Centre of Excellence in Bioinformatics, The University of Queensland, St Lucia, Brisbane, QLD 4072, Australia; 2 School of Mathematics and Statistics, The University of Melbourne, Parkville, Melbourne, VIC 3010, Australia

## Abstract

Many microbes can acquire genetic material from their environment and incorporate it into their genome, a process known as lateral genetic transfer (LGT). Computational approaches have been developed to detect genomic regions of lateral origin, but typically lack sensitivity, ability to distinguish donor from recipient, and scalability to very large datasets. To address these issues we have introduced an alignment-free method based on ideas from document analysis, term frequency-inverse document frequency (TF-IDF). Here we examine the performance of TF-IDF on three empirical datasets: 27 genomes of *Escherichia coli* and *Shigella*, 110 genomes of enteric bacteria, and 143 genomes across 12 bacterial and three archaeal phyla. We investigate the effect of *k*-mer size, gap size and delineation of groups on the inference of genomic regions of lateral origin, finding an interplay among these parameters and sequence divergence. Because TF-IDF identifies donor groups and delineates regions of lateral origin within recipient genomes, aggregating these regions by gene enables us to explore, for the first time, the mosaic nature of lateral genes including the multiplicity of biological sources, ancestry of transfer and over-writing by subsequent transfers. We carry out Gene Ontology enrichment tests to investigate which biological processes are potentially affected by LGT.

Many microbes can acquire DNA from an exogenous source (other microbes, or the environment) and maintain it for transmission to subsequent generations, either incorporated into the new host genome or stabilised on a plasmid or other extra-chromosomal element. This process, lateral genetic transfer (LGT; also known as horizontal genetic transfer), generates size and gene-content diversity among microbial genomes, and is a major driver of metabolic innovation^1^,^2^,^3^ including resistance to antibiotics^4^,^5^.

Computational approaches have been applied to detect regions of lateral origin in microbial genomes since the 1990s^6^,^7^. In the accompanying article^8^ and elsewhere^9^ we review the main biological and computational factors that make LGT detection so challenging. Briefly, there is great diversity (and little predictability) with regard to the length, source or features of the introgressed DNA. LGT events can overwrite an existing sequence, including other lateral regions, rendering the new host genome an evolutionary pastiche or mosaic. Over time, features (*e.g.* G+C content or codon usage) indicative of lateral origin will be “ameliorated” to become indistinguishable from those of the new host genome^9^,^10^,^11^. For these reasons, accurately identifying regions of lateral origin can be very challenging.

Next-generation sequencing technologies are increasingly making it possible for researchers to address large-scale questions in the biological sciences, including open questions regarding the mechanisms and impact of LGT^2^,^9^,^12^. Several computational approaches are available to detect regions of probable exogenous origin in a genome, among which tree-based methods are considered to be the gold standard^13^. Taking genes (gene families) as the units of analysis, these approaches delineate orthogroups, multiply align sets of sequences, infer gene trees and compare their topologies against that of a reference “species” tree; well-supported instances of topological incongruence are taken as *prima facie* instances of LGT^14^,^15^,^16^. Such workflows are computationally demanding, yet cannot identify recombination breakpoints in individual genomes, and often fail to resolve the direction of transfer. They can be accelerated by use of approximate methods, better matching of computational tasks to hardware, and parallelisation, but nonetheless remain slow with large datasets^17^.

For these reasons there is much interest in approaches that avoid altogether the potentially NP-hard steps of multiple sequence alignment, tree inference and tree reconciliation, while keeping track of regions of each individual genome in a manner that is agnostic to the number, size and nature of units of transfer. Alignment-free approaches have much to offer in this context. Among the main families of alignment-free approaches, those based on word counts or on substring match lengths have received the most attention^18^,^19^. The former compute a measure of similarity between two sequences based on the number or frequency distribution of matching “words” of length *k*, whereas the latter assess the length of the longest word that occurs in two sequences, or the shortest word unique to one of the sequences. In either case the match may be required to be perfect, or a defined number of mismatches may be permitted. In the simplest case, each pairwise measure can be transformed into a distance, and a matrix of such distances used as input for computing a distance tree, *e.g.* by neighbour-joining^20^,^21^,^22^. Evidence is accumulating that in phylogenetic inference *per se*, these alignment-free methods can offer acceptable performance – in certain cases better than approaches based on multiple sequence alignment – at much greater computational speed and scalability^19^. Other approaches to alignment-free sequence comparison, including methods based on compressibility^20^,^21^, nucleotide correlations^23^,^24^, gene order or recombination breakpoints^25^,^26^, have seen more-limited application. There has, however, been little exploration of how any of these alignment-free methods might be extended to other steps in an LGT workflow.

In the accompanying article^8^ we introduce TF-IDF as a scalable alignment-free approach to identify directional LGT in large molecular-sequence datasets. Variants of TF-IDF are widely used in text mining and information retrieval, for example to find important words, group and classify documents by topic, or retrieve documents that match a user query^27^,^28^. Using synthetic nucleotide-sequence data, we showed that by using TF-IDF we can detect LGT events with high precision and recall under a range of biologically realistic scenarios including different rates of deletion and nucleotide substitution^8^. We reported that TF-IDF performs well with a small empirical dataset (seven genomes of *Staphylococcus aureus*^29^) even though our target group consisted of a single sequence, presumably reducing the influence of the IDF term. The regions identified by TF-IDF as lateral matched closely with those inferred by a well-regarded method, ALFY^30^, while in addition we identified two regions not found using ALFY that include genes encoding transporters and regulators of multidrug resistance and pathogenicity^8^.

Here we explore the strengths and limitations of TF-IDF as applied to the discovery of regions of lateral origin among different-sized sets of empirical microbial genome sequences. Specifically, we investigate the effects of key parameter-value settings (*k*, and gap size *G*), and strategies for delineating, including or excluding, and subdividing groups. We consider how to interpret multiple inferred transfers into the same genome sequence, and look for evidence for overwriting. Furthermore, we determine the biological process annotations over- or under-represented among the genes we infer to have been affected by LGT, and report new LGT networks. Three empirical datasets (and variants) have been selected to illustrate a diversity of potential use cases, and address the above issues.

## Results

### Parameter values for TF-IDF analysis

**Figure 1:**
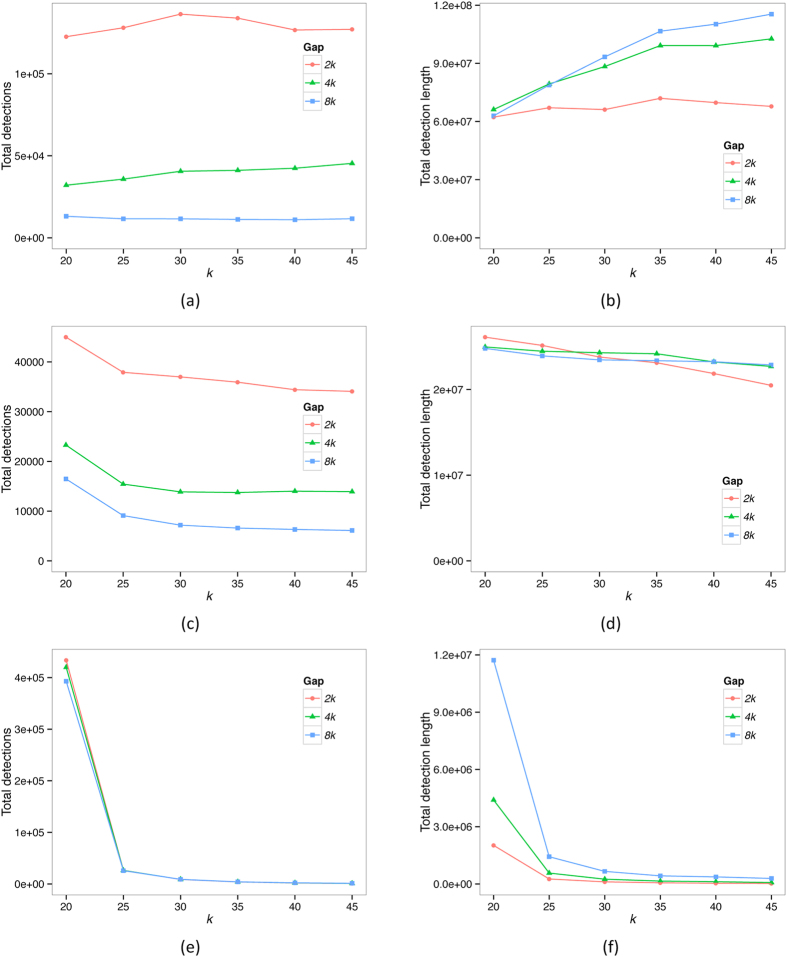
Number of regions detected as lateral, as a function of *k* and *G*. The panels on the left show total numbers of LGT detections in the ECS (panel a), EB (panel c) and BA (panel e) datasets. The panels on the right show the total length (in nucleotides) of all LGT detections in the same datasets.

To investigate the performance of TF-IDF on empirical data, we compare the number of regions identified as of potential lateral origin (Fig. 1a,c,e) and the total length of these regions (Fig. 1b,d,f) as a function of *k* and *G* in our three datasets. We examine the results in more detail for each dataset separately, and then discuss how to select suitable parameters in different situations.

#### Dataset 1 (E. coli and Shigella: ECS)

Here we use the six groups suggested by Skippington and Ragan^31^. TF-IDF presents the 27 ECS genomes as having sustained very extensive LGT from within the ECS clade itself. Inferred lateral segments hundreds of nucleotides in length are common (Supplementary Table S1), and the gaps between these segments tend to be small.

We show the dependence of the total number of lateral regions detected and the total length of all detections on *k* and *G* in Fig. 1a,b respectively. As gap size *G* increases, the total number of detections decreases sharply, indicating that many potential LGT segments are being merged together. When *k* is large, we see a corresponding rise in the total length. However, when *k* is small, the total length is relatively stable with respect to *G*, in part because the gaps between segments contain *k*-mers that are also frequent in the recipient genome’s own group, causing further proposed mergers to fail the TF hurdle. These *k*-mers can be false positives. With these considerations in mind, we set G to 2*k.* At this value, we see that both number and length of detections are relatively stable with respect to *k*. We choose the value *k* = 40, which we have shown to work well in simulations^8^.

#### Dataset 2 (enteric bacteria: EB)

The EB clade is biologically more ancient than ECS, and accordingly their genomes show smaller similarity values (Table 1). Delineating groups within the EB dataset by genus, we find fewer and shorter LGT detections than in the ECS dataset (Supplementary Table S1). As before, we see a dramatic decrease in total number of detections as *G* increases (Fig. 1c); however, the total detection length (Fig. 1d) remains relatively stable with respect to *G* at all values of *k*. This again indicates that a large number of false positive segments are being merged with increasing *G*, and thus we again set *G* = *2k*. Here there is a substantial decrease in the total number of detections as *k* increases from 20 to 25, suggesting that there are too many common *k*-mers at this value. We again choose a large value of *k* = *40* to avoid this problem.

#### Dataset 3 (bacteria and archaea: BA)

The 143 BA genomes are much less closely related among themselves, with their common biological ancestor dating nearly to origin of cellular life^32^. These genomes share many fewer identical *k*-mers than do ECS or EB (Table 1), and *k* plays a much more important role than does *G.* Because regions of inferred lateral origin in this dataset present a much weaker signal than in the previous datasets, we should set *k* to a small value in order to detect these signals. We observe (Fig. 1e,f) a precipitous drop in the both the number of detections and detection length from *k* = 20 to 25, again indicating the presence of too many common *k*-mers at the former value to make any useful detections. However, the detections are more stable for *k* 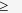
25, so we set *k* = 25. The value of *G* appears to make relatively little difference, so we again select *G* = *2k* for consistency.

We note that TF-IDF is not biased toward detecting more LGT events in larger datasets. With suitable settings of *k* and *G* (as discussed above), fewer regions of within-dataset lateral origin, totaling fewer nucleotides, are detected in EB and BA than in ECS even though they contain many more sequences. For the subsequent analyses, we fix *k* and *G* at the optimal values we have found above.

### LGT networks and effect of grouping

Next we investigate the networks of inferred LGT among the genomes in each of our datasets. TF-IDF requires that we recognise or delineate groups of sequences in the dataset; an inferred LGT event represents transfer into a genome from a donor group (other than that containing the recipient genome). Using Dataset 1, we explore the effect of different ways of delineating groups. With Datasets 2 and 3 we ask whether adding further potential donor groups affects the inference. As our results will form the basis of functional analysis (see next section), here we aggregate inferred LGT events by gene. Although genes are not units of LGT^33^,^34^, they are our link to functional annotation, notably in the GO database^35^. This mapping moreover allows us to explore, for the first time, multiple and overlapping transfers in a functional context. As intergenic regions account for only minor proportions of these genomes, we anticipate that results aggregated by gene will be substantially applicable to whole genomes as well.

#### Dataset 1 (E. coli and Shigella)

We apply two strategies for delineating groups in the ECS dataset. One uses six established phyletic groups^36^, thereby reflecting the diverse biological and physiological features that underlie the recognition of taxa in ECS. The other is explicitly phylogenetic: we cut the MRP supertree of Skippington and Ragan^31^ at basal branches to yield four groups. For details of group membership, please consult Supplementary Fig. S1. As a control, we also generate 50 pseudo-replicate groupings based on the latter.

**Figure 2:**
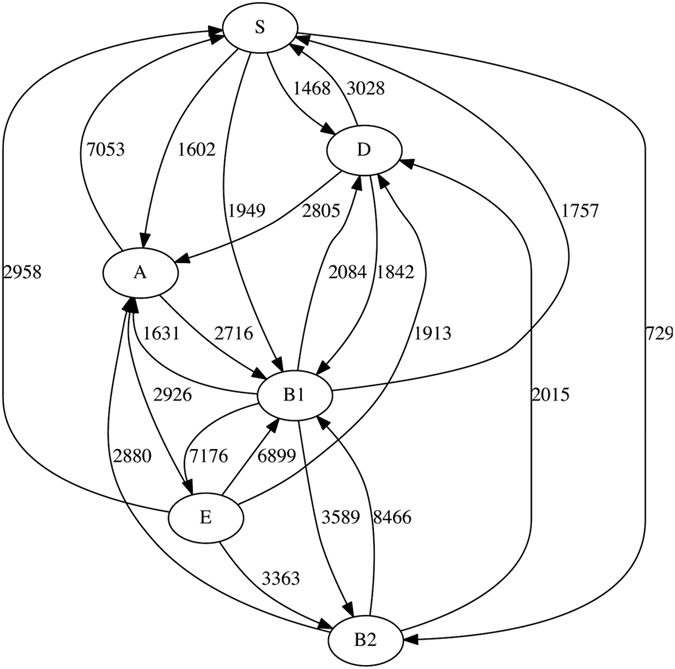
Inferred network of LGT within the ECS dataset with six biologically based groups. The numbers on each edge show the total number of genes involved in LGT events from one group into genomes in the other group.

We begin with the biological (phyletic) delineation of groups. Of the 124717 genes annotated in the 20 *E. coli* and 7 *Shigella* genomes, we infer 45412 (36.4%) to have received LGT from at least one source group. Figure 2 shows the directed LGT network connecting the groups of *E. coli* (A, B1, B2, D and E) and *Shigella* (S). Of these 27 genomes, we infer 24 to have accepted an LGT event (Table 2). Group E (*E. coli* O157:H7 EDL933 and O157:H7) has been the most-active donor group, supplying genetic material to a total of 18059 genes across all the other groups. Group E has also been the most-active recipient of LGT on a per-gene basis, with 7177 of its 10490 genes (68.4%) showing evidence of LGT, all donated from Group B1. The genome *E. coli* O157:H7 in group E is known to have acquired substantial genetic material by LGT^37^; this was notably not found by using a classical LGT detection method^31^. Group B1 (*E. coli* E24377A, 55989, SE11 and IA11) has been second most-active both as donor and recipient, donating to 16237 genes across all the other groups, and accepting LGT into 12131 of 18751 genes (64.7%), with all other groups as donors (Table 2 and Fig. 2).

**Figure 3:**
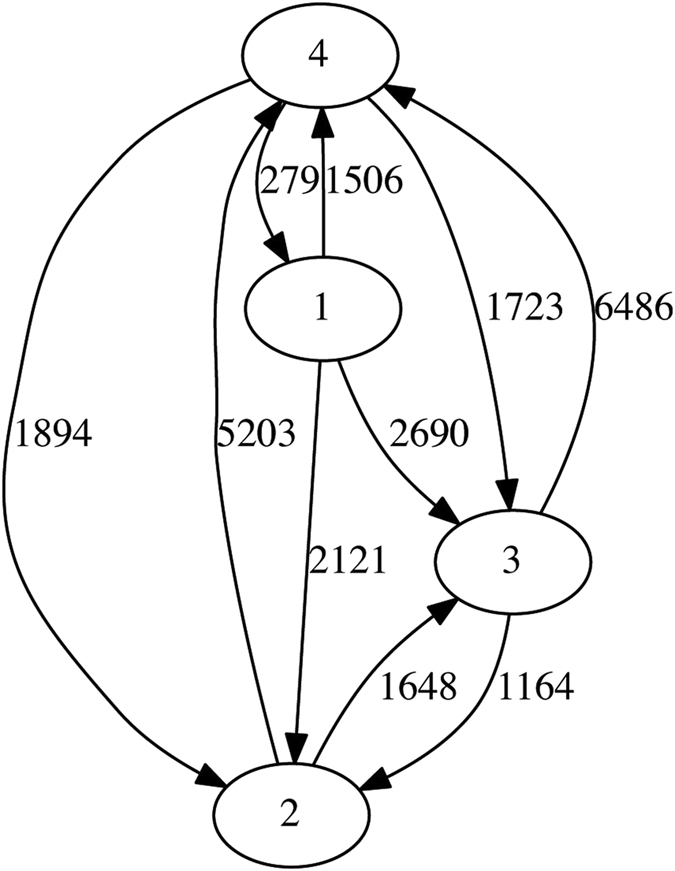
Inferred network of LGT within the ECS dataset with four phylogenetically based groups. The numbers on each edge show the total number of genes involved in LGT events from one group into genomes in the other group.

To evaluate how grouping affects the inference of LGT using TF-IDF, we delineated a different number of groups (four) using a phylogenetic criterion (see above). As physiology is not entirely orthogonal to phylogeny, the two groupings are not unrelated. Phylogenetic Group 1 includes B2 and two members of D; Group 2 encompasses E, one member of D and one *Shigella*; Group 3 has the same membership as A; and B1 and the remainder of S are merged into Group 4 (for complete lists see Supplementary Table S2). We now infer 18200 genes to have received LGT from at least one source group (Table 3), only 40.1% of the number detected in the previous grouping. The directed network is shown in Fig. 3.

The fortuitous stability of Group A or 3 (see above) allows us to make a meaningful comparison of the results between the two groupings. We infer the Group A genomes to have accepted LGT into 5841 (34.1%) of their genes, and the very same Group 3 to have accepted LGT into 4031 genes (23.5%). This difference should reflect compositional changes within the donor groups, which affects the IDF step. Conversely, group A is inferred to have affected 39571 other genes by transfer, but only 14169 genes are affected by Group 3 in the second grouping. This reflects the increased *k*-mer diversity of each recipient group, which decreases the TF threshold (that the frequency must fall below) and thus results in fewer detections. The four Group A/3 genomes show grossly similar trends between the two groupings, although details differ *e.g*. in number of genes with ≥3 LGT donor groups.

Finally, using results from TF-IDF analysis of the ECS dataset with the biological grouping, we compared the mean G + C content of the lateral genes (not just their inferred lateral regions) with that of their host genome, using a paired *t*-test. We find that G + C content is significantly higher in the inferred lateral genes; the P-value is 0.0017. Anomalous G + C content has often been used to detect transferred genes^10^,^38^.

**Figure 4:**
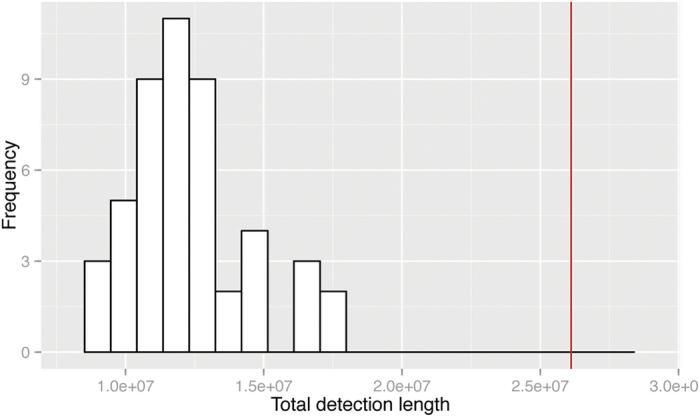
Histogram of the distribution of total length of all detections of the 50 randomly assigned replicates. The total detection length in the actual grouping (based on cutting the MRP supertree) is shown as a red line.

**Figure 5:**
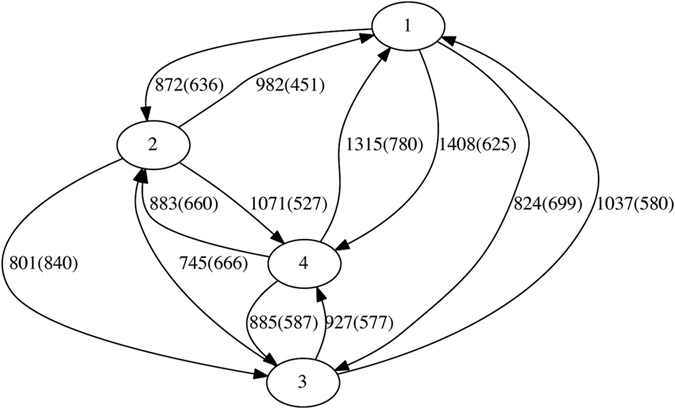
Summary of the LGT networks inferred using TF-IDF of the 50 random replicates of the ECS data. The numbers on each edge show the total number of genes involved in LGT events from one group into genomes in the other group, averaged over the 50 groupings. Numbers in parentheses are the standard deviations.

To explore the level of transfer signal in the dataset, we generate 50 randomised groupings based on the four phylogenetic groups as described above. In Fig. 4, we compare the total detection length for the randomised groups against the real grouping. The total detection length in the real grouping is much greater than for any randomised grouping; indeed it is 6.2 standard deviations above the mean. Thus we are very confident that there exists strong lateral signal in this dataset, and that the grouping we have selected is effective in showing it. In Fig. 5 we show the LGT network for the randomised groups. For seven of the twelve directional edges, we detect more genes in the actual grouping than in most (44–50) of the replicates. No LGT is found from Group 2 or 3 to Group 1 using the actual grouping information, while from Group 4 to Group 1 we inferred fewer genes than in 48 of the randomised groupings.

#### Dataset 2 (enteric bacteria)

**Figure 6:**
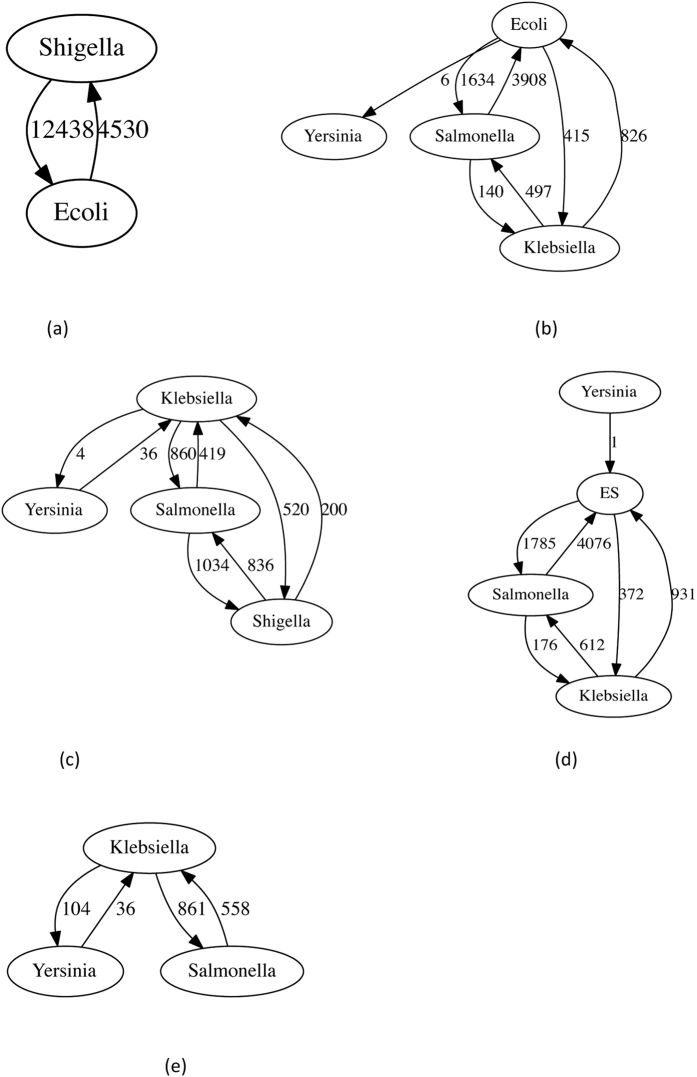
LGT networks of the EB dataset and its variants. We treat the *E. coli* and *Shigella* genomes in different ways: (**a**) assigned to separate groups, (**b**) with *Shigella* removed, (**c**) with *E. coli* removed, (**d**) combined into a single group, and (**e**) with both groups removed from the analysis.

Dataset 2a is a superset of ECS, containing additional genomes of *E. coli* and *Shigella*, plus genomes from *Klebsiella*, *Salmonella* and *Yersinia*. Naïvely taking the five genera as groups, we infer LGT only between *E. coli* and *Shigella* (Fig. 6a). This happens because the lateral signal is dominated by the 62 *E. coli* and *Shigella* genomes, which are far more similar to each other (and thus share many more identical *k*-mers) than with the remaining genera. Since by default we set the IDF threshold to the average frequency of shared *k*-mers between a sequence and a group, only the *E. coli* and *Shigella* transfers are strong enough to overcome this threshold. There are potentially several non-exclusive ways to circumvent this situation, *e.g.* by manually overriding the default use of the mean value, or reducing the number of ECS genomes or groups. Here we reduce the number of groups by alternatively merging *E. coli* and *Shigella* into a single group, keeping only one or the other, or deleting both (Table 4). Figure 6 shows the LGT networks inferred in each case. Although the actual numbers of inferred transfers (even outside *E. coli* and *Shigella*) depend strongly on how we deal with *E. coli* and *Shigella*, common trends are nonetheless apparent, *e.g.* that *Salmonella* genomes are always inferred to have accepted more LGT from *Klebsiella* than *vice-versa*, and that *Yersinia* is only weakly connected.

**Figure 7:**
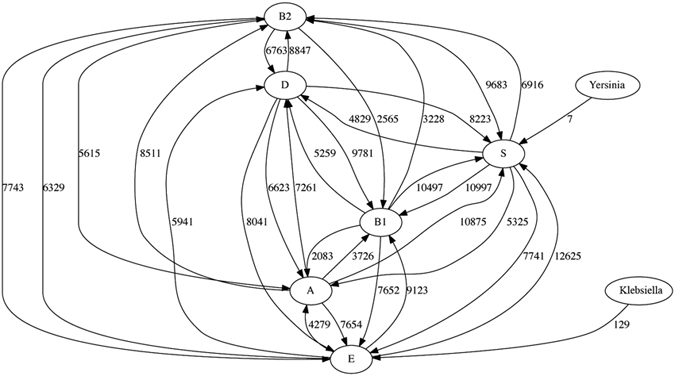
The LGT network inferred using TF-IDF from Dataset 2b, using six phyletic groups for *E. coli* and *Shigella* and grouping the remaining genomes according to genus. The numbers on each edge show the total number of genes involved in LGT events from one group into genomes in the other group.

Dataset 2 also allows us to investigate the effect of external groups (here *Klebsiella*, *Salmonella* and *Yersinia*) on inference within a clade (ECS). We generate Dataset 2b by replacing the 62 *E. coli* and *Shigella* genomes in Dataset 2a with the 27 ECS genomes as above. Using the phyletic (biological) grouping introduced above for the ECS dataset and retaining the default criterion for the IDF threshold (see previous paragraph), TF-IDF infers many more transfers within ECS (Figs 2 and 7 and Supplementary Table S3). Inclusion of the more-distantly related genomes has lowered the mean value of elements in the relationship matrix, thereby allowing many more regions within the ECS genomes to exceed the IDF threshold. Even with the additional TF filter, which remains unchanged, this results in a great increase in the number of transfers. As might be expected, all transfers detected in the ECS dataset by itself are still detected. Interestingly, this increase is non-uniform across the ECS subgraph: in every case where we inferred no LGT from one group into another when only the ECS dataset was examined, we found abundant LGT after adding the three additional genera.

As mentioned above, another option is to manually override the default use of the mean *k*-mer frequency value as the IDF threshold. When we set the threshold from the ECS dataset as the IDF threshold in Dataset 2b, we infer exactly the same genes in the ECS genomes to have accepted LGT from an ECS donor group. That is, presence or absence of external groups does not affect the performance of TF-IDF beyond their effect on the IDF threshold. We consider this further in the Discussion.

#### Dataset 3 (bacteria and archaea)

**Figure 8:**
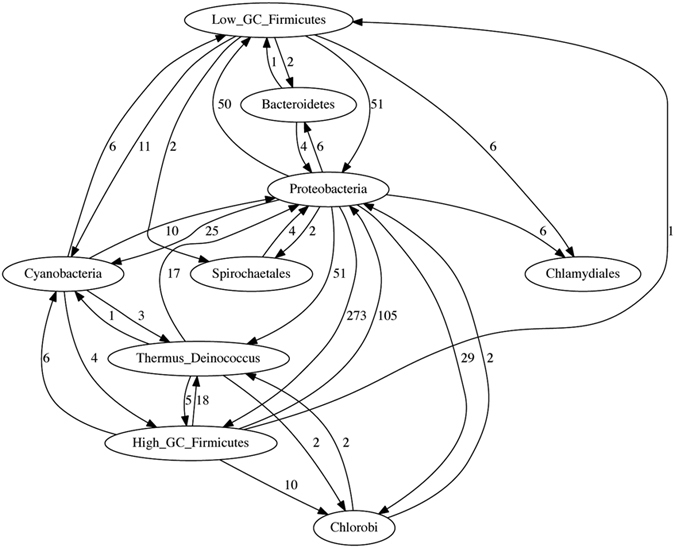
The LGT network inferred for the BA dataset grouped by phylum. The numbers on each edge show the total number of genes involved in LGT events from one group into genomes in the other group.

The 143-genome Bacteria and Archaea (BA) dataset allows us to examine the effect of within-group heterogeneity on inference using TF-IDF. Here we delineate groups taxonomically by phylum (15 phyla) or alternatively by class (31 classes). Grouping the genomes by phylum, we infer 686 genes as affected by LGT, many fewer than in the smaller but less-divergent previous datasets. Indeed, we infer no inter-phylum LGT involving the archaeal phyla (Crenarchaeota, Euryarchaeota, Nanoarchaeota) or three of the bacterial phyla (Aquificales, Planctomycetes, Thermotogales), presumably for the reason indicated above for Datasets 2a and 2b: potential matches fail to pass the IDF threshold. Of the nine remaining bacterial phyla, eight are inferred to have been both donors and recipients, while one (Chlamydiales) has been a recipient only (Fig. 8). The highest-activity pathways (“highways”^14^) lie between Proteobacteria and High-G + C Firmicutes (378 genes affected), followed by those between Proteobacteria and Low-G + C Firmicutes (101 genes). Two phyla represented by one genome each, Thermus/Deinococcus and Chlorobi, contribute 13.9% and 6.3% of total inter-phylum LGT; if more sequences had been included, these groups might be recognised as even more-active in inter-phylum LGT.

**Figure 9:**
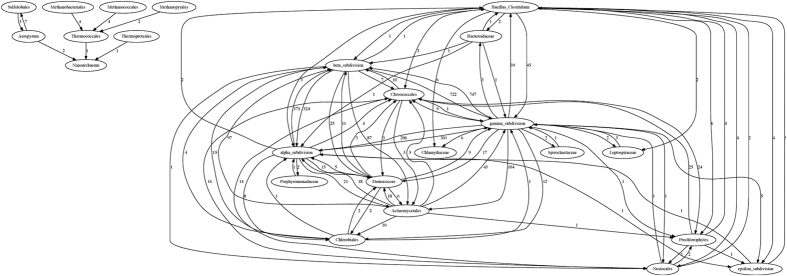
The LGT network inferred for the BA dataset grouped by class. The numbers on each edge show the total number of genes involved in LGT events from one group into genomes in the other group.

When we alternatively group the 143 genomes into 31 classes (Fig. 9), the number of genes inferred to have accepted inter-class LGT increases nearly five-fold to 3043. We infer 24 lateral genes among eight archaeal classes with a ninth class, Archaeoglobales, silent to inter-class LGT. As above, no LGT is detected between archaea and bacteria. This grouping divides Proteobacteria into four subdivisions (α, β, γ, ε) at class level; genomes of the former three are rich in inferred lateral genes, whereas the ε subdivision is relatively silent. In accordance with our phylum-level analysis, the Bacillus/Clostridium class and Actinomycetales (from High-G+C Firmicutes) are inferred to have engaged in LGT with genomes across the subdivisions of Proteobacteria. By contrast, and in contradiction to earlier reports, we infer no LGT involving the *Thermotoga*^39^ or *Aquifex*^40^ genomes. This may be due to features of our dataset *e.g.* the number, size, balance, composition and cohesion of groups, and/or the phylogenetic distinctiveness of these genomes (see Discussion and conclusions).

Using a dataset of 657 bacterial and archaeal genomes and a multi-step LGT inference approach based on anomalous G+C content and phylogenetic discordance, Popa *et al*.^41^ identified 4700 genes of inferred lateral origin. Nine of these were also identified as lateral in our TF-IDF analysis of the 143-genome dataset, although with different inferred donors. Our comparison (Supplementary Section 2 and Supplementary Tables S4, S5, S6, S7) indicates that at least at this phyletic scale, TF-IDF provides access to LGT events spanning broader phyletic distances than does the approach of Popa *et al*.^41^.

### Multiple donor groups and superimposed transfers

In the ECS dataset we observe a large number of transfers; correspondingly, we find many instances in which a gene is inferred to have accepted genetic material laterally from more than one donor group. This is especially prevalent in the phyletic grouping of six groups, whereas the phylogenetic grouping (four groups) contains fewer transfers as observed above. Here we look more closely at genomes which contain genes with multiple donors, to determine if we can untangle the sources of multiple transfer.

There are two possible explanations for such instances. One is that the gene is truly a mosaic, having accepted multiple transfers in the past. However, an alternative explanation is that there was only one transfer, but it was more ancient. For example, if a sequence is inferred to have accepted genetic material from groups G1 and G2, then it is possible that instead there was a single ancestral transfer from an ancestor of G1 and G2. This explanation is parsimonious only if G1 and G2 are closely related (*i.e.* monophyletic, or adjacent on the phylogenetic tree) and the events are inferred to affect overlapping regions on the genome. If either of these conditions is not met, it is more likely that more than one transfer event has occurred.

In Table 5, we examine the relative frequencies of possible ancestral transfers in eighteen ECS genomes. For ease of analysis, we consider only genes which are inferred to have accepted material from exactly two donor groups. Of the 2240 such genes in *E. coli* K12 W3110, K12 MG1655, HS and IAI1, which contain monophyletic relationships in their donor-group pairs, we observe 18446 events into these genes, forming 6549 overlapping regions. Of these overlapping regions, the donor groups are monophyletic in 1869 cases. Thus there is considerable evidence for both ancient transfers and mosaicism. However, ancient transfers (identified in this way) represent only 22–30% of overlapped events in these four genomes. For the other genomes no monophyletic overlapping regions are found, *i.e.* most overlaps may be the result of multiple lateral events. This is the first time a computational method has given us broad accessibility to data that can indicate the presence of these phenomena.

### Biological process enrichment

To determine the frequencies at which different sorts of proteins are implicated in our LGT detections, we extracted protein-name annotations from the corresponding GenBank files (Table 6). The most-frequent name annotation is in LGT events is *hypothetical protein*, followed by *membrane protein*, *transcriptional regulator* and *transporter*, protein types known to be exchanged among bacteria^42^,^43^. To further investigate the biological processes affected by LGT, we carried out functional enrichment tests (see Methods), selecting a false discovery rate of 0.05 as significance threshold. Here we present a general discussion of biological processes over- or under-represented in the datasets; full lists of terms are given in Supplementary Tables S8–S10.

Among the ECS genomes, enrichment analysis identifies metabolite and trans-membrane transport, carbohydrate metabolic processes, and small-molecule biosynthesis and catabolism as particularly over-represented as inferred targets of LGT; at least 42 of the 50 most over-represented terms refer specifically to such processes. By contrast, the 21 most under-represented terms refer to transposition, genetic recombination, translation, or metabolism of peptides or nitrogenous compounds.

Within the Enteric Bacteria (EB) dataset, enrichment of terms can depend on how we group the *E. coli* and *Shigella* genomes. When 62 *E. coli* and *Shigella* genomes are combined into a single group, biological processes related to translation, nitrogen-compound and RNA biosynthesis, and viruses dominate the most over-represented functions, while trans-membrane transport and polysaccharide metabolism are under-represented. Removing all *E. coli* and *Shigella* genomes, only the *E. coli* genomes, or only the *Shigella* genomes, does not greatly affect this picture. When the *E. coli* and *Shigella* genomes are retained but grouped separately, the TF-IDF analysis is dominated by LGT between these groups (Fig. 6a); viral processes including entry into and release from host cells, and extracellular (lipo)polysaccharide biosynthesis, come to the fore among over-represented processes, while translation, transposition, and purine and ribose metabolism are now under-represented. These results illustrate how grouping can affect the functional interpretation of LGT in bacterial genomes.

With the BA dataset grouped by phylum, relatively few genes are inferred to have accepted LGT (above). Thirty-five processes are found to be over-represented (see Supplementary section 4), with *translational elongation* (GO:0006414) being by far the most significant. No under-represented process passes our FDR threshold. Grouping instead by class, diverse metabolic processes appear as over-represented, while only two processes appear as (slightly) under-represented.

## Discussion and Conclusions

TF-IDF is an alignment-free method for the detection of regions of exogenous origin in molecular sequences. Based on the content of *k*-mers in a specific dataset, the method can identify regions of exogenous origin in a sequence, and their inferred donor groups within the dataset, with high efficiency and effectiveness^8^. Here we apply TF-IDF on three empirical microbial-genome datasets of different sizes and sequence diversity to explore the advantages and limitations of this method. We systematically varied two key parameter-value settings (for word length *k* and gap size *G*), and investigated how the delineation of groups affects the performance of TF-IDF.

Our results indicate that it may not be possible to identify a value of *k* optimal for all datasets. However, for these microbial genome datasets, the distribution of shared 12-mers helps us to select *k.* Within the ECS genomes, the vertical components of which share a relatively recent common ancestor, the proportion of identical 12-mers is relatively high (median >60%) and a longer *k* (35 ≤ *k* ≤ 50) supports high-confidence detections while not missing too many real LGTs. By contrast, in the highly divergent BA dataset in which most genomes share <30% identical 12-mers pairwise, almost no LGT is detected at k ≥ 40. To ensure adequate LGT signal in such a dataset, *k* must be set smaller (20 ≤ *k* ≤ 30). However, at *k* ≤ 15, *k*-mers are too frequently matched pairwise at random, leading to an unacceptable level of false positives. In general, larger values of *k* are appropriate for high-similarity datasets, and shorter *k* for low-similarity data.

*G* determines how aggressively nearby lateral *k*-mers are consolidated into a single region. Given a sufficient density of such *k*-mers, a larger *G* causes intervening non-lateral regions to be merged into the consolidated region. This can cause some false positive regions to be detected by TF-IDF. At shorter *G*, the total number of detections increases without greatly affecting total detection length. Thus shorter *G* is preferred for precise delineation of lateral segments. In most cases, *G* = 2*k* is a satisfactory option.

Apart from *k* and *G*, TF-IDF is also sensitive to how groups are recognised within the dataset. Many more transfers were inferred within the ECS dataset when six, rather than four, groups were recognised. It is difficult to disentangle the effects of group number, size, composition and phylogenetic cohesion, but we use the fortuitous stability of Group A/3 to argue that both TF and IDF terms can contribute to this sensitivity. We further demonstrate that the presence or absence of external groups does not affect the performance of TF-IDF beyond their effect (*via* the constituent sequences) on the IDF threshold. These results emphasise that as implemented here, TF-IDF is deterministic and is self-tuning to the dataset.

We suspect that these effects manifest so strongly in ECS Groups E and B1 because these lineages have been particularly active in LGT. Analysing a 64-genome superset of our ECS dataset, Lukjancenko *et al*.^44^ find that members of Group E contribute almost half of the new gene families, while our four Group B1 strains contribute aggressively to the rise in pan-genome size. Additional effects arising from variation in gene content may contribute further.

TF-IDF delivers the most power when applied to a sequence dataset with high within-group similarity but uniformly low between-group similarity. However, group structure can be arbitrary in real-life cases. Our results with the ECS subset of Dataset 2 illustrate strategies for dealing with uneven or unbalanced data. The substantial loss of LGT signal when we assign sequences randomly to groups strongly indicates that groups should be delineated so as to capture the underlying phylogenetic structure where possible. This may not always be possible, as in our BA dataset, where *Thermotoga* and *Aquifex* are represented by single genomes of uncertain but relatively distant phylogenetic relationship. Other strategies for delineating groups can be imagined, but lie outside the scope of the present study.

It has long been considered that lateral transfers from different donor groups can be superimposed in the recipient genome, yielding mosaic or pastiche genes^45^,^46^. We have now demonstrated this in the ECS dataset. Most genes that have accepted LGT have done so in multiple events, often from different donor groups. Where group structure reflects evolutionary history and neighbouring genomic regions are inferred to have been donated by groups adjacent on the tree, the transfer may have been ancestral. Taking a gene-centric approach restricted (for simplicity of analysis) to genes with only two inferred lateral origins, we find that a modest proportion (22–30%) might best be explained by ancestral transfer. To our knowledge, this is the first systematic computational study of multiple or overlapping origins in empirical genome-scale data.

We mapped genes containing the inferred lateral regions to Gene Ontology (GO) terms using BLAST2GO then applied enrichment tests, identifying a wide range of biological processes as preferentially affected by LGT. Many processes known to be shared laterally are indeed over-represented, although others (including e.g. transposition) are under-represented, whether as a consequence of their actual distribution in the dataset, or their presumed origin from a donor group not represented in the dataset.

Our inference that genes annotated as involved in *translational elongation* (GO:0006414) in the BA dataset, and in *translation* (GO:0006412) in the EB dataset, are overrepresented among the LGT sets bears comment, as “informational” functions are considered less-susceptible to LGT than “operational” genes *e.g*. those involved in cellular transport or metabolism^42^. Closer examination reveals that (1) substantial subsets of our LGT-enrichment sets annotated with *translational elongation* (in BA) or *translation* (in EB) are not core informational genes, or indeed informational genes at all, but appear in our lists *via* secondary annotations *e.g*. involving specialised regulatory relationships; (2) many informational genes^42^ are well-known to be susceptible to LGT; (3) even “core” informational genes are sometimes transferred laterally; and (4) in a few cases, core informational genes that we infer as lateral have features or properties (*e.g.* constraints, domains, paralogs, phyletic distributions) that could indicate a lateral history, or help explain why a lateral history has gone unrecognised by classical methods (for details see Supplementary material). Further, *translation* is over-represented when the 62 *E. coli* and *Shigella* genomes are combined into a single group; when they all are removed from the analysis; or when only *E. coli*, or only *Shigella*, are removed. However, when we include these *E. coli* and *Shigella* genomes but group them separately, *translation* becomes under-represented. That is, the LGT “translation” signal is being driven from parts of the dataset other than the *E. coli-Shigella* axis, and is completely overshadowed (indeed driven to under-representation) by the (much stronger) signal from the mostly non-translational transfers between *E. coli* and *Shigella*.

For each dataset, the groups (nodes) and inferred transfers (edges) constitute the LGT network. Each of these three networks exhibits one or more densely connected regions (subgraphs), as well as nodes that are more-weakly connected or unconnected. The lack of connection between archaea and bacteria in the BA dataset is a case in point: far fewer transfers are inferred between archaea and bacteria than internally among archaea, or internally among bacteria. In the TF-IDF analysis of Dataset 2a, *Yersinia* remains almost unconnected to other genera; this illustrates that even among LGT-active groups, some genera can remain inactive.

In summary, our results demonstrate that TF-IDF can be applied on diverse empirical genome-scale datasets, resulting in the inference of inter-group directional LGT and providing first steps toward the systematic reconstruction of multiple and superimposed transfer events. These inferred transfers affect a broad range of biological processes, including many already known or suspected to be affected by LGT. Future work will explore whether and how the settings of *k* and *G* affect topological features of the inferred LGT networks, hence our interpretation of lateral biology in microbial communities and the biosphere.

## Methods

### Datasets

From our earlier simulation study^8^ we know that the performance of TF-IDF can be affected by how groups are delineated within a dataset, and by the divergence of sequences within a group. If sequences within groups are similar to one another (expected mutations up to 0.16/nucleotide) and the groups are dissimilar from one another (expected mutations between neighbouring groups above 0.2/nucleotide), the boundaries between groups are clear and TF-IDF can achieve high precision (>80%) and recall (>90%)^8^. Here, we select three empirical datasets that differ in number of sequences and divergence among sequences, to explore the performance of TF-IDF under a range of biologically realistic situations. Table 7 shows general information (number of sequences, sequence lengths and G + C content) on these datasets, while further information is presented in the following paragraphs.

#### Dataset 1

*Escherichia coli* and *Shigella* (abbreviated hereafter as ECS), represented by 20 and 7 genomes respectively. Here and elsewhere^47^ the *Shigella* genomes are resolved as one or more lineages within the genus *Escherichia*. Some genomes within ECS are known to be rich in regions of inferred lateral origin^47^. Using alignment-based methods, we have previously shown that lateral transfer of protein-coding regions within ECS is biased by phylogeny (*i.e.* genetic relatedness and/or sequence similarity) more than by environment^31^, whereas the distribution of small RNAs has been affected more by gene loss than by LGT^48^. For the present work we recognise groups within ECS in two alternative ways: (1) by cutting the MRP supertree^31^ at certain levels (see Supplementary Fig. S1), or (2) by using recognized phyletic groups^36^. These approaches yield four and six groups respectively.

#### Dataset 2a

110 genomes from the Enterobacteriaceae (53 *Escherichia*, 9 *Shigella*, 9 *Klebsiella*, 22 *Salmonella* and 17 *Yersinia*), here abbreviated EB. Among these *Escherichia*, *Shigella, Klebsiella* and *Salmonella* are considered relatively susceptible to LGT. Strains of *Yersinia* harbour plasmids that encode genes of probable lateral origin^49^,^50^ but our datasets exclude plasmid sequences. *Yersinia* appears not to be naturally competent^51^ and although its main chromosome shows evidence of pathogenicity islands, their genes match sequences outside the Enterobacteriaceae^52^ and thus would not be recognised as lateral in our analyses of Dataset 2. We recognise each genus as a separate group except for *E. coli* and *Shigella*, which we treat in different ways (see Results).

#### Dataset 2b

These 75 genomes constitute a subset of Dataset 2a (pruned to 20 *E. coli* and 7 *Shigella*) and a superset of Dataset 1 (addition of 58 genomes from the other genera). We expect to see the same LGT detections within *E. coli* and *Shigella* as in the ECS dataset when the threshold is the same. Together, Datasets 2a and 2b allow us to explore the effects of group inclusion/exclusion (of groups other than ECS) and subdivision (ECS).

#### Dataset 3

143 genomes across 12 bacterial and 3 archaeal phyla, abbreviated here as BA. This dataset allows us to explore the effects of phyletic breadth, degree of sequence divergence, unbalanced group size and disruptive genomes on LGT inference. This dataset has been well-explored in our group using classical alignment-based (and some novel) methods for more than ten years^14^,^33^,^34^; MRP^53^ and 16S rRNA reference trees are available. This dataset moreover offers a more-general (less-biased) selection of Gene Ontology (GO) Biological Process (BP)^35^ annotations than do specialist datasets dominated by human and animal pathogens (our Datasets 1 and 2).

**Figure 10:**
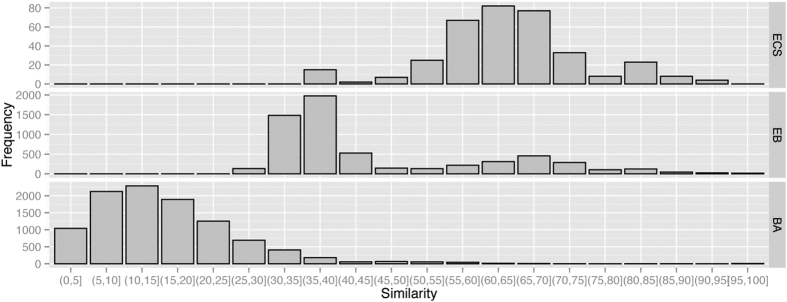
Distribution of 12-mer frequencies in the ECS, EB and BA datasets. The *x*-axis shows binned proportions of 12-mers shared pairwise over all genomes in each dataset; the left *y*-axis shows frequencies (counts) of 12-mers, and the right *y*-axis gives the name of the dataset.

These datasets span a variety of evolutionary divergences. Information on the divergence among a dataset is important for setting the parameters of TF-IDF; however, typical approaches based on alignments are time-consuming and do not scale well with increasing number of sequences. To quantify this variation, we thus compute a rough measure of sequence similarity by calculating the percentage of identical 12-mers shared between each pair of sequences. Summary information is presented in Table 1. The distribution of similarities is shown in Fig. 10; here we see that as expected, the ECS genomes are most similar pairwise, the EB genomes are more divergent (with a small bimodality consistent with the ECS subset) and the BA genomes the most divergent, with most sequence pairs sharing fewer than 30% of their 12-mers.

### TF-IDF and parameterisation

In this study we apply the TF-IDF method we devised in previous research^8^. TF-IDF is an alignment-free method that detects LGT by the relative frequencies of *k*-mers in pre-determined groups. The method proceeds in four steps:

1. Extract all unique *k*-mers in a dataset and build a *k*-mer dictionary of the dataset.
2. IDF: we count the identical *k*-mers between each sequence and each group other than its own. A relationship matrix *R* is built in which rows are genomes, columns are groups, and individual elements count the number of identical *k*-mers shared between a sequence and a group. For consistency across group sizes and genome lengths, we normalise these counts by dividing by the number of genomes in the group (column), and by the number of nucleotides in the genome (row). We then compute the mean over all elements in *R*. If the value of an element exceeds the mean, the corresponding genome potentially contains lateral events (segments) donated by that group.
3. For each genome with potential transfers from a donor group, we construct potential LGT segments by amalgamating all neighbouring *k*-mers in the genome which also appear in that group. These segments are further merged by joining all segments which are separated by an amount less than a threshold, which we refer to as gap size (*G*).
4. TF: if the average frequency of all lateral *k*-mers in a candidate LGT segment is lower than the average frequency of all *k*-mers in the group containing that genome, then that segment is considered to have arisen by LGT.

In this work we vary word length *k* and gap size *G* (see Table 8). Based on the results of our previous study^8^, we limit *k* to the range 20–45; when *k* < 20 many detected events are false positives, while at *k* ≥ 50 common *k*-mers become too rare, resulting in decreased performance. Values of *G* were selected to cover a biologically reasonable range of granularity consistent with computational feasibility.

For the ECS dataset, we also vary group composition in order to study its effect on inference using TF-IDF. We recognise groups in two ways as described above; in addition, we also generate 50 randomised groupings patterned on the first grouping (into four groups by phylogeny) by allocating each sequence to a group chosen at random, while preserving the number of sequences in each group. By doing this we generate a control set in which vertical inheritance signal is greatly attenuated, and against which we can compare our actual grouping. The total detection length based on actual groups (generated by cutting the MRP tree) is significantly higher than from the random replicates.

### Gene Ontology mapping and enrichment tests

For each recipient genome, our TF-IDF analysis outputs a list of regions (coordinates) inferred to be of lateral origin, and the inferred donor group of each. To identify the biological functions affected by these regions, we map these coordinates to genes annotated in the host genome (as given in the NCBI.ffn and.gbk files). For both biological and statistical reasons, we examine only relatively long regions: biologically we are interested only in LGT events with potential to have functional consequence, while statistically we seek to minimise false positives and noise. Thus, a gene is considered lateral only if it contains at least one segment which is longer than a given threshold. These thresholds (given in Table 9) are selected on different datasets to be close to the average length of all LGT detections in that dataset. This accounts for the variation in sequence diversity among the datasets.

For each dataset we used blastp^54^ at E ≤ 10^−5^ to match protein-coding regions annotated in all genomes to the Swiss-Prot database^55^. Genes were distinguished by GI number and position. Gene Ontology (GO) terms associated with the matches were retrieved using BLAST2GO^56^,^57^ version 3.3.1 (mapping and annotation functions) from GO database version b2g_may15, yielding the background database for enrichment testing. We then submit a list of genes implicated as recipients of LGT, querying this list against the entire database. Regardless of the number of inferred lateral regions or donor groups involved, each gene is counted only once. We use a two-tailed Fisher’s exact test with a false discovery rate (FDR) of 0.05. This yields a list of GO Biological Process (BP) annotations which are over- and under-represented in the test set^58^.

## Additional Information

**How to cite this article**: Cong, Y. *et al*. Exploring lateral genetic transfer among microbial genomes using TF-IDF. *Sci. Rep.* **6**, 29319; doi: 10.1038/srep29319 (2016).

## Supplementary Material

Supplementary Information

Supplementary Table S8

Supplementary Table S9

Supplementary Table S10

## Figures and Tables

**Table 1:** Summary of genome similarity (percentage of pairwise shared 12-mers) for the three datasets.

Dataset	Minimum %	Maximum %	Mean %	Standard deviation %
ECS	37.15	94.5	64.06	10.58
EB	29.08	99.99	45.43	15.65
BA	0.4243	99.42	15.94	10.44

For abbreviations see text.

**Table 2:** Numbers of lateral genes with single or multiple donors in each genome within the ECS dataset.

Group	Organism	Number of genes	Number of lateral genes	Donor groups	Number of lateral genes with 1 donor	Number of lateral genes with 2 donors	Number of lateral genes with ≥3 donors
D	*E. coli* SMS 3 5	4744	0	–	–	–	–
D	*E. coli* IAI39	4725	1469	S	1469	–	–
D	*E. coli* UMN026	4878	2930	S, B1, B2	1004	769	1157
S	*Shigella flexneri* 5 8401	4336	1030	E, A, B1	855	120	55
S	*Shigella flexneri* 2a	4053	1142	D, E, A	650	288	204
S	*Shigella flexneri* 2a 2457T	4385	1188	E, A, B1	880	158	150
S	*Shigella sonnei* Ss046	4563	1842	D, E, A, B1	638	626	578
S	*Shigella boydii* Sb227	4391	1859	D, E, A, B1	974	538	347
S	*Shigella boydii* CDC 3083 94	4532	1280	A, B1	1093	187	–
S	*Shigella dysenteriae*	4063	1300	A	1300	–	–
E	*E. coli* O157 : H7	5204	3685	B1	3685	–	–
E	*E. coli* O157 : H7 EDL933	5286	3492	B1	3492	–	–
A	*E. coli* K12 substr W3110	4213	1211	S, E, B1	1050	117	44
A	*E. coli* K12 substr MG1655	4140	2045	D, E, B1, B2	927	465	653
A	*E. coli* HS	4366	2585	D, S, E, B1, B2	891	667	1027
A	*E. coli* C ATCC 8739	4434	0	–	–	–	–
B1	*E. coli* E24377A	4729	2945	S, E, A, B2	1469	1068	412
B1	*E. coli* 55989	4953	3108	S, E, A, B2	1526	1081	501
B1	*E. coli* SE11	4684	2918	S, E, A, B2	1448	1049	421
B1	*E. coli* IAI1	4385	3157	D, S, E,A, B2	1161	991	1005
B2	*E. coli* 0127 H6 E2348 69	4809	1561	E, B1	1120	448	–
B2	*E. coli* 536	4542	1183	E, B1	855	328	–
B2	*E. coli* CFT073	4897	0	–	–	–	–
B2	*E. coli* ED1a	5012	1230	S, E	941	305	–
B2	*E. coli* UTI89	4827	563	B1	563	–	–
B2	*E. coli* S88	4688	1088	E, B1	801	287	–
B2	*E. coli* APECO1	4878	592	B1	592	–	–

The genomes are grouped into six groups following a phyletic criterion (see text).

**Table 3:** Numbers of lateral genes with single or multiple donors in each genome within the ECS dataset.

Group	Organism	Number of genes	Number of lateral genes	Donor groups	Number of lateral genes with 1 donor	Number of lateral genes with 2 donors	Number of lateral genes with ≥3 donors
2	*E. coli* UMN026	4878	2122	1	2122		
4	*Shigella flexneri* 5 8401	4336	923	2, 3	680	243	
4	*Shigella flexneri* 2a	4053	590	2, 3	445	145	
4	*Shigella flexneri* 2a 2457T	4385	999	2, 3	674	325	
4	*Shigella sonnei* Ss046	4563	988	2, 3	688	300	
4	*Shigella boydii* Sb227	4391	1314	2, 3	848	466	
4	*Shigella boydii* CDC 3083 94	4532	1244	2, 3	879	365	
2	*Shigella dysenteriae*	4063	1363	3, 4	498	865	
2	*E. coli* O157 : H7 EDL933	5286	832	4	832		
3	*E. coli* K12 substr W3110	4213	875	2, 4	596	279	
3	*E. coli* K12 substr MG1655	4140	1255	1, 4	860	395	
3	*E. coli* HS	4366	1901	1, 2, 4	948	587	366
4	*E. coli* E24377A	4729	694	2, 3	495	199	
4	*E. coli* 55989	4953	1193	1, 2, 3	670	361	162
4	*E. coli* SE11	4684	683	2, 3	457	226	
4	*E. coli* IAI1	4385	944	1, 2, 3	507	300	137
1	*E. coli* ED1a	5012	280	4	280		

The genomes are grouped into four groups by cutting the MRP supertree (see text).

**Table 4:** Variants of the EB dataset based on treatment of the *E. coli* and *Shigella* groups.

Ways of grouping	Group information	Number of sequences
Combine *E. coli* and *Shigella*	*Yersinia*, *E. coli* + *Shigella*, *Salmonella*, *Klebsiella*	110
Keep only *E. coli*	*Yersinia*, *E. coli*, *Salmonella*, *Klebsiella*	92
Keep only *Shigella*	*Yersinia*, *Shigella*, *Salmonella, Klebsiella*	57
No *E. coli* or *Shigella*	*Yersinia*, *Salmonella*, *Klebsiella*	48

**Table 5:** Numbers of inferred lateral genes with two donors in the ECS dataset, and the phyletic relationship of the donors.

Genome name	Lateral genes with two donors	Number of overlaps (pairwise)	Number monophyletic	Number non-monophyletic	Proportion monophyletic
*E. coli* K12 substr W3110	117	223	50	173	22.4%
*E. coli* K12 substr MG1655	465	1514	423	1091	27.9%
*Shigella flexneri* 2a	288	709	0	709	0
*Shigella flexneri* 2a 2457T	158	361	0	0	0
*Shigella sonnei* Ss046	626	1564	0	1564	0
*Shigella boydii* Sb227	538	1247	0	1247	0
*E. coli* 536	328	522	0	522	0
*Shigella flexneri* 5 8401	120	259	0	259	0
*E. coli* HS	667	1885	564	1321	29.9%
*E. coli* E24377A	1068	2272	0	2272	0
*Shigella boydii* CDC 3083 94	187	277	0	277	0
*E. coli* SE11 (#20)	1049	2250	0	2250	0
*E. coli* 0127 H6 E2348 69	448	670	0	670	0
*E. coli* IAI1	991	2927	832	2095	28.4%
*E. coli* S88	287	413	0	413	0
*E. coli* ED1a	305	431	0	431	0
*E. coli* 55989	1081	2344	0	2344	0
*E. coli* UMN026	769	2445	0	2445	0

**Table 6:** Top 25 protein names (as extracted from the GenBank files) inferred to be affected by an LGT event in the ECS dataset.

Counts	Protein name
11944	hypothetical protein
1426	membrane protein
1056	transcriptional regulator
615	transporter
454	oxidoreductase
265	transposase
251	LysR family transcriptional regulator
232	tail protein
192	two-component system response regulator
190	lipoprotein
182	two-component system sensor histidine kinase
162	diguanylate cyclase
158	hydrolase
144	AraC family transcriptional regulator
141	MFS transporter
135	ABC transporter ATP-binding protein
125	porin
124	peptide ABC transporter permease
121	putative DNA-binding transcriptional regulator
115	protease
105	fimbrial protein
104	sensor histidine kinase
102	multidrug ABC transporter ATP-binding protein
100	glycosyl transferase
95	ABC transporter permease

**Table 7:** General description of the datasets investigated in this research.

Name	Number of sequences	Mean length	Range of lengths	Mean G + C content (%)	Range of G + C content (%)
ECS	27	4906162	4369232–5528445	50.76	50.39–51.33
EB	110	4920079	3976195–6097032	51.03	47.00–57.68
BA	143	3011345	490885–9105828	45.67	22.48–72.12

**Table 8:** Range of parameter values investigated with the TF-IDF method. *k* is the size of *k*-mers.

Parameter	Parameter values
Word length *k*	20–45 in steps of 5
Gap size *G*	2*k*, 4*k*, 8*k*

**Table 9:** Thresholds for mapping LGT segments to genes.

Dataset	Thresholds for laterally transferred genes
ECS	>500 contiguous *k*-mers (500 + *k* − 1 nt)
EB	>100 contiguous *k*-mers (100 + *k* − 1 nt)
BA	>10 contiguous *k*-mers (10 + *k* − 1 nt)

The thresholds are selected by means of all detections in three empirical datasets.
