# Supplementary material for: Exploring lateral genetic transfer among microbial genomes using TF-IDF
Source: Sci Rep. 2016 Jul 25;6:29319. doi: 10.1038/srep29319 (PMC4958990; doi:10.1038/srep29319)
Supplement: Supplementary Information [file srep29319-s1.pdf]

# Supplementary Material

# Exploring lateral genetic transfer among microbial genomes using TF-IDF

Yingnan Cong

Yao-ban Chan

Mark A. Ragan

**Overview of Supplementary Material:**

**Section 1. Supplementary material for ECS (*E. coli* and *Shigella* genomes) dataset, and for comparison among our datasets**

Supplementary Tables S1-S3 and Supplementary Figure S1

**Section 2. Comparison between TF-IDF analysis of our BA (bacteria and archaea) dataset with grouping by class, and results reported by Popa *et al*. (2011) on a 657-genome bacteria and archaea dataset.**

Supplementary Tables S4-S7

**Section 3. Index of sheet names and contents for Excel spreadsheets**

These spreadsheets (Supplementary Tables S8-S10), reporting Gene Ontology enrichment results for our three main datasets, are too large to be displayed within this file. They are available as separate files.

**Section 4. Supplementary material for Gene Ontology enrichment tests**

Supplementary Tables S11-S12

**Section 1. Supplementary material for ECS (*E. coli* and *Shigella* genomes) dataset, and for comparison among our datasets**

**Table S1.** Summary of lengths of the inferred lateral segments, showing the mean, median first quartile, and median third quartile lengths of all inferred segments.

| Dataset | Mean | Median | First quartile | Third quartile |
|---|---|---|---|---|
| ECS | 550.70 | 269 | 113 | 622 |
| EB | 116.22 | 56 | 8 | 104 |
| BA (phylum level) | 7.188 | 3 | 2 | 6 |
| BA (class level) | 9.84 | 4 | 2 | 8 |

**Table S2.** Group assignments for ECS genomes into six or four groups (see text and Figure S1).

| Six groups | Four groups | Organism |
|---|---|---|
| D | 1 | *E. coli* SMS 3 5 |
| D | 1 | *E. coli* IAI39 |
| D | 2 | *E. coli* UMN026 |
| S | 2 | *Shigella dysenteriae* |
| S | 4 | *Shigella flexneri* 5 8401 |
| S | 4 | *Shigella flexneri* 2a |
| S | 4 | *Shigella flexneri* 2a 2457T |
| S | 4 | *Shigella sonnei* Ss046 |
| S | 4 | *Shigella boydii* Sb227 |
| S | 4 | *Shigella boydii* CDC 3083 94 |
| E | 2 | *E. coli* O157:H7 |
| E | 2 | *E. coli* O157:H7 EDL933 |
| A | 3 | *E. coli* K12 substr W3110 |
| A | 3 | *E. coli* K12 substr MG1655 |
| A | 3 | *E. coli* HS |
| A | 3 | *E. coli* C ATCC 8739 |
| B1 | 4 | *E. coli* E24377A |
| B1 | 4 | *E. coli* 55989 |
| B1 | 4 | *E. coli* SE11 |
| B1 | 4 | *E. coli* IAI1 |
| B2 | 1 | *E. coli* 0127 H6 E2348 69 |
| B2 | 1 | *E. coli* 536 |
| B2 | 1 | *E. coli* CFT073 |
| B2 | 1 | *E. coli* ED1a |
| B2 | 1 | *E. coli* UTI89 |
| B2 | 1 | *E. coli* S88 |
| B2 | 1 | *E. coli* APECO1 |

**Table S3**. Summary of lateral regions inferred among the 27 ECS genomes in Dataset 2b (*i.e.* when these 27 ECS genomes replace the 52 *E. coli* and *Shigella* genomes in the EB dataset, and *Salmonella*, *Klebsiella* and *Yersinia* genomes are present).

| **Group** | **Organism** | **Number of genes** | **Number of lateral genes** | **Donor groups** |
|---|---|---|---|---|
| D | *E. coli* SMS 3 5 | 4744 | 3346 | - |
| D | *E. coli* IAI39 | 4725 | 3212 | S |
| D | *E. coli* UMN026 | 4878 | 3615 | S, B1, B2 |
| S | *Shigella flexneri* 5 8401 | 4336 | 1989 | E, A, B1 |
| S | *Shigella flexneri* 2a | 4053 | 1850 | D, E, A |
| S | *Shigella flexneri* 2a 2457T | 4385 | 2091 | E, A, B1 |
| S | *Shigella sonnei* Ss046 | 4563 | 2436 | D, E, A, B1 |
| S | *Shigella boydii* Sb227 | 4391 | 2388 | D, E, A, B1 |
| S | *Shigella boydii* CDC 3083 94 | 4532 | 2347 | A, B1 |
| S | *Shigella dysenteriae* | 4063 | 2236 | A |
| E | *E. coli* O157:H7 | 5204 | 4489 | B1 |
| E | *E. coli* O157:H7 EDL933 | 5286 | 4570 | B1 |
| A | *E. coli* K12 substr W3110 | 4213 | 2534 | S, E, B1 |
| A | *E. coli* K12 substr MG1655 | 4140 | 2580 | D, E, B1, B2 |
| A | *E. coli* HS | 4366 | 2983 | D, S, E, B1, B2 |
| A | *E. coli* C ATCC 8739 | 4434 | 3183 | - |
| B1 | *E. coli* E24377A | 4729 | 3628 | S, E, A, B2 |
| B1 | *E. coli* 55989 | 4953 | 3836 | S, E, A, B2 |
| B1 | *E. coli* SE11 | 4684 | 3616 | S, E, A, B2 |
| B1 | *E. coli* IAI1 | 4385 | 3512 | D, S, E,A, B2 |
| B2 | *E. coli* 0127 H6 E2348 69 | 4809 | 3021 | E, B1 |
| B2 | *E. coli* 536 | 4542 | 2702 | E, B1 |
| B2 | *E. coli* CFT073 | 4897 | 2844 | - |
| B2 | *E. coli* ED1a | 5012 | 2925 | S, E |
| B2 | *E. coli* UTI89 | 4827 | 2681 | B1 |
| B2 | *E. coli* S88 | 4688 | 2709 | E, B1 |
| B2 | *E. coli* APECO1 | 4878 | 2781 | B1 |

**Figure S1.** MRP supertree of the ECS dataset [1], further annotated to show the two ways we group the 27 ECS genomes: by a biological criterion (into Groups D, S, E, A, B1 and B2) or by cutting the supertree on deep branches (into Groups 1, 2, 3 and 4).

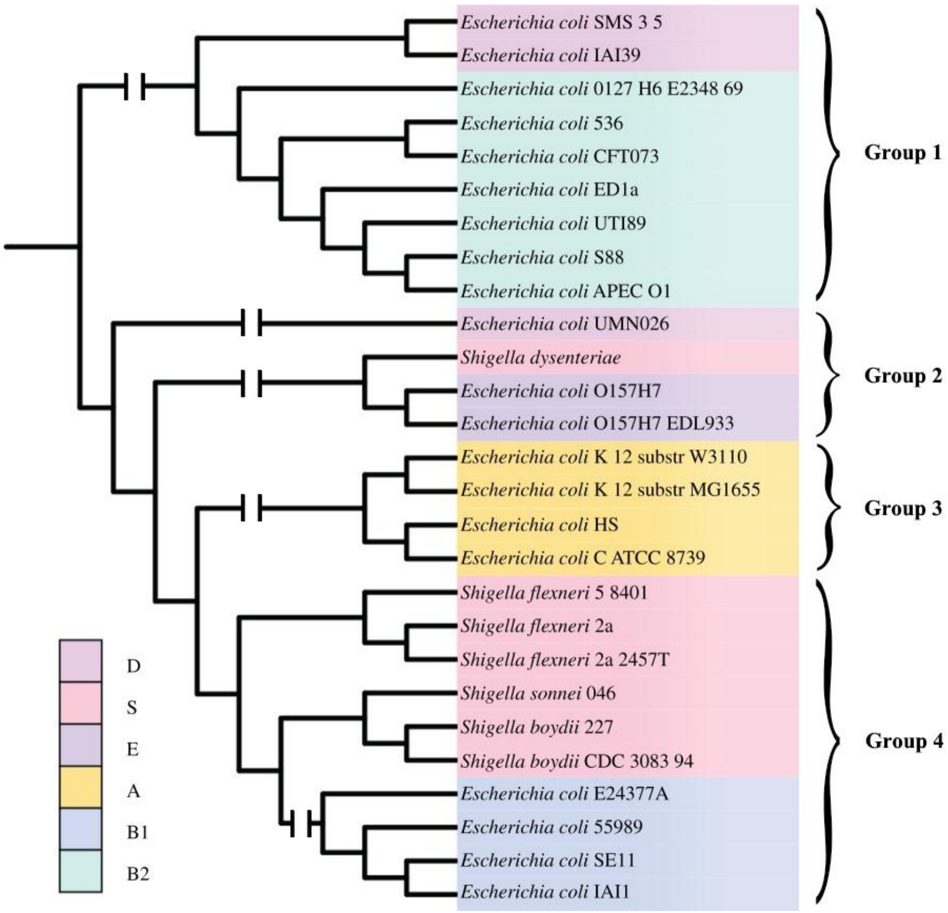



[1] Skippington, E. & Ragan, M. A. Phylogeny rather than ecology or lifestyle biases the construction of *Escherichia coli-Shigella* genetic exchange communities. *Open Biology* **2**, 120112 (2012).

**Section 2. Comparison between TF-IDF analysis of our BA (bacteria and archaea) dataset with grouping by class, with results reported by Popa *et al*. (2011) on a 657-genome bacteria and archaea dataset.**

Popa and colleagues [1] analysed a dataset of 657 bacterial and archaeal genomes (sets of individual genes) using an approach that allows genes to be identified as lateral. In some cases (about 7% of the total) they can infer the direction of transfer. Lists of their genomes (Genome_list_dLGT.txt) and inferred lateral genes (dLGT-data.txt) are available as Supplemental Material [1]. Here we compare these lists with the corresponding results from our TF-IDF analysis of Dataset 3 (143 bacterial and archaeal genomes) analysed at class level, and report on overlaps.

**POPA [1]:**
Groups: 17 groups (mostly classes & phyla)
Genomes: 657
Genes: 2,129,548
Lateral genes: 52,621 (from dLGT-data.txt)
Unique lateral GI numbers: 41,392 (from dLGT-data.txt)
Unique lateral inter-generic GI numbers (donors and recipients): 5819
Genomes contributing these 5819 inter-generic lateral GIs: 317
Unique lateral inter-generic GI numbers (recipients only): 4700
Genomes contributing these 4700 recipient GIs: 277

**OUR DATASET 3 (BACTERIAL & ARCHAEAL GENOMES):**
Level-3 groups: 31 classes
Genomes: 143
Genes: 390,801
Unique GI numbers: 375,468
Lateral events: 3623 ($k$ = 25, $G$ = 2$k$ = 50)
TF-IDF between-class lateral recipient GIs: 3043 (coverage threshold: see Main text, Table 9)
Genomes contributing these 3043 recipient GIs: 100

**OVERLAP DATA:**
Genomes in both POPA and BA: 40 (genus/species/strain descriptors identical or nearly identical)*
Group overlap: substantial overlap or similarity (see Tables S5 and S6)
GIs common to POPA 41,392 and BA 375,468: 4513 (POPA laterals in BA)
GIs common to POPA 41,392 and BA 3403: 81 (POPA laterals in BA laterals)
GIs common to POPA 5819 and BA 375,468: 800 (POPA inter-generic laterals in BA)
GIs common to POPA 5819 and BA 3043: 9 (POPA inter-generic laterals in BA laterals)

*Because Popa *et al*. do not report genome versions, precise (version) identity would have to be reverse-engineered from GI lists (which themselves are incomplete). However, the numbers of matched GI numbers suggest that at least half of the 40 genomes appearing in both the POPA and BA lists are represented by the same, or nearly identical, versions.

Thus when the two methods are applied to large, similarly diverse (bacteria plus archaea) datasets, they detect about the same density of unique “long-distance” lateral recipient GIs:

POPA: 4700 inter-generic recipients / 317 contributing genomes
TF-IDF: 3623 inter-class recipients / 100 contributing genomes

However, the POPA “long-distance” transfers are almost always within-class or even closer (*Escherichia* and *Shigella* are almost certainly the same genus). This is apparent from Figure 2A of Popa *et al*. [1], where very few clusters (connected components) encompass nodes of different colours. By contrast, all the “long-distance” transfers inferred using TF-IDF are necessarily (given the way we delineate groups in this case) between-class.

As illustrated by the nine inferred recipient genes above, the appearance of a gene in LGT lists from these two approaches does not imply that the same (or a compatible) lateral event has been inferred. A decade ago one of us [2] pointed out that different “surrogate” (non-phylogenetic) methods may agree less often than expected under a purely stochastic model. G+C-based methods preferentially identify relatively recent transfer events [3]. The method employed by Popa *et al*. [1] is a hybrid (G+C plus phylogenetic) method, but its initial screening step is based on G+C content.

We conclude that TF-IDF provides access to LGT events spanning broader phyletic distances than does the approach of Popa *et al*. [1].

**REFERENCES FOR SECTION 2**

[1] Popa O, Hazkani-Covo E, Landan G, Martin W & Dagan T. Directed networks reveal genomic barriers and DNA repair bypasses to lateral gene transfer among prokaryotes. *Genome Res*. 21: 599-601 (2011).

[2] Ragan MA. On surrogate methods for detecting lateral gene transfer. *FEMS Microbiol. Lett*. 201: 187-191 (2001).

[3] Ragan MA, Harlow TJ & Beiko RG (2006) Do different surrogate methods detect lateral genetic transfer events of different relative ages? *Trends Microbiol*. 14: 4-8 (2006).

**Table S4. Nine GIs common to POPA 5819 and BA 3043 datasets (see above).**

```
1
RECIPIENT     16272575 Haemophilus influenzae Rd KW20 (Gamma-proteobacteria)
GENE          NP_438792.1 Elongation factor Tu
DONOR POPA    875 Actinobacillus_pleuropneumoniae_L20 (Gamma-proteobacteria)
DONOR TF-IDF  Bacillus/clostridium

2
RECIPIENT     16762890 Salmonella enterica subsp. enterica serovar Typhi str.
              CT18 (Gamma-proteobacteria)
GENE          NP_458507.1 B12-dep homocysteine-N5-methyltetrahydrofolate
              transmethylase
DONOR POPA    822 Klebsiella_pneumoniae_MGH_78578 (Gamma-proteobacteria)
DONOR TF-IDF  Deinococcus

3
RECIPIENT     16763372 Salmonella enterica subsp. enterica serovar Typhi str.
              CT18 (Gamma-proteobacteria)
GENE          NP_458989.1 ABC transporter ATP-binding protein
DONOR POPA    818 Citrobacter_koseri_ATCC_BAA-895 (Gamma-proteobacteria)
DONOR TF-IDF  Alpha-proteobacteria

4
RECIPIENT     16767822 Salmonella enterica subsp. enterica serovar
              Typhimurium str. LT2 (Gamma-proteobacteria)
GENE          NP_463437.1  ABC transporter ATP-binding protein
DONOR POPA    818 Citrobacter_koseri_ATCC_BAA-895 (Gamma-proteobacteria)
DONOR TF-IDF Alpha-proteobacteria

5
RECIPIENT     27467230 Staphylococcus epidermidis ATCC 12228 (Low-GC
              Firmicutes)
GENE          NP_763867.1 Elongation factor Tu
DONOR POPA    324 Enterococcus_faecalis_V583 (Low-GC Firmicutes)
DONOR TF-IDF Gamma-proteobacteria

6
RECIPIENT     30018378 Bacillus cereus ATCC 14579 (Low-GC Firmicutes)
GENE          NP_830009.1 Elongation factor Tu
DONOR POPA    281 Lysinibacillus_sphaericus_C3_41 (Low-GC Firmicutes)
DONOR TF-IDF Gamma-proteobacteria

7
RECIPIENT     30260299 Bacillus anthracis str. Ames (Low-GC Firmicutes)
GENE          NP_842676.1 Translation elongation factor Tu
DONOR POPA    281 Lysinibacillus_sphaericus_C3_41 (Low-GC Firmicutes)
DONOR TF-IDF  Gamma-proteobacteria

8
RECIPIENT     33593471 Bordetella pertussis Tohama I (Beta-proteobacteria)
GENE          NP_881115.1 Isocitrate dehydrogenase
DONOR POPA    57 Ralstonia_eutropha_H16 (Beta-proteobacteria)
DONOR TF-IDF  Gamma-proteobacteria

9
RECIPIENT     56480411 Shigella flexneri 2a str. 301 (Gamma-proteobacteria)
GENE          NP_709501.2  DNA gyrase subunit B
DONOR POPA    801 Escherichia_coli_O157H7 (Gamma-proteobacteria)
DONOR TF-IDF  Beta-proteobacteria
```

(In addition, 56480411 appears twice as a donor in POPA, in each case donating to an Escherichia coli O157:H7 strain)

**TABLE S5.** Genomes in our BA data, grouped at Level 3 (by class).

| | | |
|---|---|---|
| Crenarchaeota: | | |
| | 1 | Aeropyrum (1 genome) |
| | 2 | Sulfolobales (2 genomes) |
| | 3 | Thermoproteales (1 genome) |
| Euryarchaeota: | | |
| | 4 | Archaeoglobales (1 genome) |
| | 5 | Halobacteriales (1 genome) |
| | 6 | Methanobacteriales (1 genome) |
| | 7 | Methanococcales (1 genome) |
| | 8 | Methanopyrales (1 genome) |
| | 9 | Methanosarcinales (2 genomes) |
| | 10 | Thermococcales (3 genomes) |
| | 11 | Thermoplasmales (2 genomes) |
| Nanoarchaeota: | | |
| | 12 | Nanoarchaeum (1 genome) |
| Aquificales: | | |
| | 13 | Aquificaceae (1 genome) |
| Bacteroidetes: | | |
| | 14 | Bacteroidaceae (1 genome) |
| | 15 | Porphyromonadaceae (1 genome) |
| Chlamydiales: | | |
| | 16 | Chlamydiaceae (7 genomes) |
| Chlorobi: | | |
| | 17 | Chlorobiales (1 genome) |
| Cyanobacteria: | | |
| | 18 | Chroococcales (4 genomes) |
| | 19 | Nostocales (1 genome) |
| | 20 | Prochlorophytes (3 genomes) |
| High G+C Firmicutes: | | |
| | 21 | Actinomycetales (12 genomes) |
| Low G+C Firmicutes: | | |
| | 22 | Bacillus/Clostridium group (34 genomes) |
| Planctomycetes: | | |
| | 23 | Planctomycetales (1 genome) |
| Proteobacteria: | | |
| | 24 | alpha subdivision (9 genomes) |
| | 25 | beta subdivision (8 genomes) |
| | 26 | epsilon subdivision (5 genomes) |
| | 27 | gamma subdivision (33 genomes) |
| Spirochaetales: | | |
| | 28 | Leptospiraceae (1 genome) |
| | 29 | Spirochaetaceae (2 genomes) |
| Thermotogales: | | |
| | 30 | Thermotoga (1 genome) |
| Thermus/Deinococcus group: | | |
| | 31 | Deinococcus (1 genome) |

**TABLE S6.** Genome numbers in groups from Popa *et al*. (2011), file “dLGT-data.txt”

| | |
|---|---|
| 59 | Actinobacteria |
| 89 | Alphaproteobacteria |
| 125 | Bacilli |
| 8 | Bacteroidetes |
| 71 | Betaproteobacteria |
| 6 | Chlamydiae |
| 4 | Chlorobi |
| 7 | Chloroflexi |
| 31 | Clostridia |
| 7 | Crenarchaeota |
| 29 | Cyanobacteria |
| 4 | Deinococcus-Thermus |
| 16 | Deltaproteobacteria |
| 15 | Epsilonproteobacteria |
| 16 | Euryarchaeota |
| 203 | Gammaproteobacteria |
| 12 | Mollicutes |
| 9 | Spirochaetes |
| 4 | Thermotogae |

**TABLE S7.** Strain names identical, or nearly identical, between the Popa *et al.* 657-genome list and ours. Close similarity of names does not guarantee identical assemblies or annotation versions (hence identical GI lists); conversely, in a few cases strain designations were changed or abbreviated, disguising potentially similar records. Our comparison of GIs was not pre-filtered through this name list, so full disambiguation of strain designators is not necessary for the purposes of this Supplementary analysis.

Bacillus_anthracis_Ames
Bacillus_cereus_ATCC_14579
Bacteroides_thetaiotaomicron_VPI-5482
Bordetella_bronchiseptica_RB50
Bordetella_parapertussis_12822
Bordetella_pertussis_TohamaI
Bradyrhizobium_japonicum_USDA110
Brucella_melitensis_16M
Brucella_suis_1330
Chlorobium_tepidum_TLS
Chromobacterium_violaceum_12472
Enterococcus_faecalis_V583
Escherichia_coli_CFT073
Escherichia_coli_O157:H7
Escherichia_coli_O157:H7_EDL933
Haemophilus_ducreyi_35000HP
Lactococcus_lactis_lactis
Mesorhizobium_loti_MAFF303099
Neisseria_meningitidis_MC58
Neisseria_meningitidis_Z2491
Nitrosomonas_europaea_ATCC_19718
Nostoc_sp._PCC_7120
Oceanobacillus_iheyensis_HTE831
Pasteurella_multocida_Pm70
Salmonella_typhimurium_LT2
Salmonella_enterica_Typhi_Ty2
Shigella_flexneri_2a_2457T
Sinorhizobium_meliloti_Rm1021
Staphylococcus_aureus_MW2
Staphylococcus_aureus_Mu50
Staphylococcus_aureus_N315
Streptococcus_agalactiae_2603V/R
Streptococcus_agalactiae_NEM316
Streptococcus_pneumoniae_R6
Streptococcus_pneumoniae_TIGR4
Vibrio_vulnificus_CMCP6
Vibrio_vulnificus_YJ016
Wigglesworthia_brevipalpis_Str.
Yersinia_pestis_CO92
Yersinia_pestis_KIM

**Section 3. Index of sheet names and contents for Excel spreadsheets**

**Table S8.** GO enrichment results on the ECS dataset (Supplementary Table S8: two sheets).

| Sheet name | Description of results |
|---|---|
| Enrich_27_OVER | GO terms over-represented in six phyletic groups |
| Enrich_27_UNDER | GO terms under-represented in six phyletic groups |

**Table S9.** GO enrichment results on the BA dataset (Supplementary Table S9: ten sheets).

| Sheet name | Description of results |
|---|---|
| E_S_o | GO terms over-represented, *E. coli* and *Shigella* grouped separately |
| E_S_u | GO terms under-represented, *E. coli* and *Shigella* grouped separately |
| Ecoli_o | GO terms over-represented, *Shigella* genomes removed |
| Ecoli_u | GO terms under-represented, *Shigella* genomes removed |
| Shigella_o | GO terms over-represented, *E. coli* genomes removed |
| Shigella_u | GO terms under-represented, *E. coli* genomes removed |
| No_E_S_o | GO terms over-represented, *E. coli* and *Shigella* genomes removed |
| No_E_S_u | GO terms under-represented, *E. coli* and *Shigella* genomes removed |
| ES_combined_o | GO terms over-represented, *E. coli* and *Shigella* combined into one group |
| ES_combined_u | GO terms under-represented, *E. coli* and *Shigella* combined into one group |

**Table S10**. Enrichment test results of BA dataset (Supplementary Table S10: three sheets).

| Sheet name | Description of results |
|---|---|
| Enrich_143_L2_OVER | GO terms over-represented, genomes grouped by phylum |
| Enrich_143_L3_OVER | GO terms over-represented, genomes grouped by class |
| Enrich_143_L3_UNDER | GO terms under-represented, genomes grouped by class |

## Section 4. Supplementary material for Gene Ontology enrichment tests

### Section 4.1 GO:0006414 translational elongation: 35 genes affected by LGT in BA dataset

2 ABC transporter ATP-binding protein
1 alanyl-tRNA ligase (synthetase)
2 leucyl-tRNA ligase (synthetase)
2 valyl-tRNA ligase (synthetase)
4 elongation factor G
21 elongation factor Tu
3 GTP-binding protein LepA

Rivera *et al*. [1] identified as "informational" genes as those functioning in translation (including tRNA synthetases), transcription and (DNA) replication, as well as homologs of vacuolar ATPases and GTPases. The following year the same group [2] included almost the same categories (omitting *replication*), and pointed to the translational and transcriptional complexes as examples. In the former they identified initiation, elongation (EF-Tu, EF-Ts, EF-G) and termination factors, ribosomal proteins, rRNAs, tRNAs and mRNAs, as well as "nongene products such as ions, small molecules such as GTP, GDP, etc., and membranes".

These informational genes were considered less-susceptible to LGT [2]. This idea has persisted, although one subsequent study found no LGT bias between informational and operational genes [3], and another study found the bias limited to the "translation, ribosomal structure, and biogenesis" category once correction was made for connectivity bias [4]. Another study found translational genes to be the functional category for which within-bacteria LGT is the MOST frequent [5]. Functional category J (translation, ribosomal structure and biogenesis) shows strong net-like relationships among some although not all bacteria [6, Figure 4J].

Not all informational genes are "resistant" to LGT. Genes encoding aminoacyl-tRNA ligases (synthetases) are well-known to be susceptible to LGT [5,7,8], specifically including the three types we found: alanyl [9], leucyl [5,10,11] and valyl [5,7,12,13] ligases.

ABC transporter subunits, including the ATP-binding protein, are likewise well-known to be susceptible to LGT [14-17].

By contrast, the "core" elongation factors EF-G and EF-Tu are often considered resistant to LGT because the deeper branches of their phylogenetic trees agree with the corresponding 16S rRNA tree. However, recent studies have added quite a lot of nuance to this generalisation. Two large systematic studies of evolutionary dynamics in the EF-G protein family [18,19] identify four [19] or five [18] classes of EF-G paralogs, several of which have been affected by gene duplication, LGT and loss. Using somewhat different data, these authors identified about 14 instances of ancestral or more-recent LGT of EF-G paralogs involving $\alpha$-, $\beta$- and $\gamma$-proteobacteria,

actinomycetes, cyanobacteria and spirochaetes; other LGT events were considered possible. Some LGT events turned up in both studies [18,19]; others were supported by presence/absence of indels [19]. Using TF-IDF, we infer that EF-G genes in four genomes (of 143 in our BA dataset) have accepted LGT: one *Staphylococcus aureus*, one *Streptococcus*, one chlamydia (all transferred from Proteobacteria) and *Deinococcus* (transferred from high-G+C Firmicutes); see Table S4 below. The latter genome has been particularly accepting of LGT [5,20,21].

Using TF-IDF, we infer that 21 EF-Tu genes in 18 of these 143 genomes have been affected by LGT. In two $\gamma$-proteobacterial genomes (*Haemophilus influenzae* and *Vibrio parahaemolyticus*), both copies of EF-Tu are inferred to have accepted LGT from the Low-G+C Firmicutes, while both copies in *Deinococcus* have accepted LGT from Proteobacteria. In all, our 21 inferred LGT events involve transfer from the low-G+C Firmicutes into $\gamma$- (four) or $\varepsilon$-proteobacterial genomes (one), or from Proteobacteria into a low- (seven) or a high-G+C Firmicute, or a member of Chlamydia, Cyanobacteria, *Deinococcus* or Fusobacteria (Table S11). The involvement of Firmicutes in all but four of these 21 inferred events is notable, and recalls the results of Ke *et al*. [22] with 17 species of the low-G+C firmicute *Enterococcus*: in all 11 species with two copies of EF-Tu, the *tufB* copy had arisen laterally, likely in a single event, whereas there was no evidence of LGT in the six species in which EF-Tu is single-copy. Ke *et al*. [22] mention potentially similar situations in the low-G+C firmicute *Clostridium*, and in the high-G+C firmicute *Steptomyces*. We also note evidence for homologous recombination affecting the EF-Tu ortholog EF-1$\alpha$ in archaea [23], and for LGT being responsible for the distribution of the EF-1$\alpha$-like factor EFL among eukaryotes [24].

Finally, our list also includes the highly conserved "fourth EF", leader peptidase A (LepA), which is present in bacteria and almost all eukaryotes, but absent from archaea. We are aware of only a single report examining its phylogeny [25]. No evidence was found for inter-kingdom LGT; taxon sampling within Bacteria was limited and bootstrap support modest (and/or not shown), but the topology of the LepA branch gives no reason to suspect the involvement of LGT.

## References for Section 4.1


[1] Rivera, M.C., Jain, R., Moore, J.E. & Lake, J.A. Genomic evidence for two functionally distinct gene classes. *Proc. Natl Acad. Sci. USA* **95**, 6239-6244 (1998).

[2] Jain, R., Rivera, M.C. & Lake, J.A. Horizontal gene transfer among genomes: the complexity hypothesis. *Proc. Natl Acad. Sci. USA* **96**, 3801-3806 (1999).

[3] Nakamura, Y., Itoh, T., Matsuda, H. & Gojobori, T. Biased biological functions of horizontally transferred genes in prokaryotic genomes. *Nat. Genet*. **36**, 760-766 (2004).

[4] Cohen, O., Gophna, U. & Pupko, T. The complexity hypothesis revisited: connectivity rather than function constitutes a barrier to horizontal gene transfer. *Mol. Biol. Evol.* **28**, 1481-1489 (2011).

[5] Kanhere, A. & Vingron, M. Horizontal gene transfers in prokaryotes show differential preferences for metabolic and translational genes. *BMC Evol. Biol*. **9**, Art. 9 (2009).

[6] Puigbò, P., Wolf, Y.I. & Koonin, E.V. The tree and net components of prokaryote evolution. *Genome Biol. Evol*. **2**, 745-756 (2010).
[7] Wolf, Y.I., Aravind, L., Grishin, N.V. & Koonin, E.V. Evolution of aminoacyl-tRNA synthetases – analysis of unique domain architectures and phylogenetic trees reveals a complex history of horizontal gene transfer events. *Genome Res.* **9**, 689-710 (1999).
[8] Woese, C.R., Olsen, G.J., Ibba, M. & Söll, D. Aminoacyl-tRNA synthetases, the genetic code, and the evolutionary process. *Microbiol. Mol. Biol. Rev*. **64**, 202-236 (2000).
[9] Boussau, B., Guéguen, L. & Gouy, M. Accounting for horizontal gene transfers explains conflicting hypotheses regarding the position of Aquificales in the phylogeny of Bacteria. *BMC Evol. Biol*. **8**, Art. 272 (2008).
[10] Dohm, J.C., Vingron, M. & Staub, E. Horizontal gene transfer in aminoacyl-tRNA synthetases including leucine-specific subtypes. *J. Mol. Evol*. **63**, 437-447 (2006).
[11] Andam, C.P., Harlow, T.J., Papke, R.T. & Gogarten, J.P. Ancient origin of the divergent forms of leucyl-tRNA synthetases in the Halobacteriales. *BMC Evol. Biol*. **12**, Art. 85 (2012).
[12] Brown, J.R. Ancient horizontal gene transfer. *Nat. Rev. Genet*. **4**, 121-132 (2003).
[13] Adato, O., Ninyo, N., Gophna, U & Snit, S. Detecting horizontal gene transfer between closely related taxa. *PLoS Comp. Biol*. **11**, Art. e1004408 (2015).
[14] Nelson, K.E., *et al*. Evidence for horizontal gene transfer between Archaea and bacteria from genome sequence of *Thermotoga maritima*. *Nature* **399**, 323-329 (1999).
[15] De Ruggiero, J. *et al*. Evidence of recent lateral gene transfer among hyperthermophilic archaea. *Mol. Microbiol*. **38**, 684-693 (2000).
[16] Noll, K.M., Lapierre, P., Gogarten, J.P. & Nanavati, D.M. Evolution of *mal* ABC transporter operons in the Thermococcales and Thermotogales. *BMC Evol. Biol*. **8**, Art. 7 (2008).
[17] Meehan, C.J. & Beiko, R.G. Lateral gene transfer of an ABC transporter complex between major constituents of the human gut microbiome. *BMC Microbiol.* **12**, Art. 248 (2012).
[18] Atkinson, G.C. & Baldauf, S.L. Evolution of elongation factor G and the origins of mitochondrial and chloroplast forms. *Mol. Biol. Evol*. **28**, 1281-1292 (2011).
[19] Margus, T., Remm, M. & Tenson, T. A computational study of elongation factor G (EFG) duplicated genes: diverged nature underlying the innovation on the same structural template. *PLoS ONE* **6**, Art. e22789 (2011).
[20] Makarova, K.S. *et al*. Genome of the extremely radiation-resistant bacterium *Deinococcus radiodurans* viewed from the perspective of comparative genomics. *Microbiol. Mol. Biol. Rev.* **65**, 44-79 (2001).
[21] Yuan, M. *et al*. Genome sequence and transcriptome analysis of the radioresistant bacterium *Deinococcus gobiensis*: insights into the extreme environmental adaptations. *PLoS ONE* **7**, Art. e34458 (2012).
[22] Ke *et al*. Evidence for horizontal gene transfer in evolution of elongation factor Tu in Enterococci. *J. Bacteriol.* **182**, 6913-6920 (2000).
[23] Inagaki, Y., Doolittle, W.F., Baldauf, S.L. & Roger, A.J. Lateral transfer of an EF-1$\alpha$ gene: origin and evolution of the large subunit of ATP sulfurylase in Eubacteria. *Proc. Natl Acad. Sci. USA* **103**, 4528-4533 (2006).
[24] Kamikawa, R., Inagako, Y. & Sako, Y. Direct phylogenetic evidence for lateral transfer of elongation factor-like gene. *Proc. Natl Acad. Sci. USA* **105**, 6965-6969 (2008).
[25] Qin, Y. *et al*. The highly conserved LepA is a ribosomal elongation factor that back-translocates the ribosome. *Cell* **127**, 721-733 (2006).

**Table S11. Recipient genomes and donor groups for elongation factors inferred for our BA dataset.**

**EF-G**
*Staphylococcus aureus* [Low-G+C Firmicutes] from Proteobacteria
*Streptococcus* [Low-G+C Firmicutes] from Proteobacteria
*Chlamydophila* [Chlamydiae] from Proteobacteria
*Deinococcus* from High-G+C Firmicutes

**EF-Tu**
*Haemophilus* [Gamma-proteobacteria] (two species) from Low-G+C Firmicutes
*Pseudomonas* [Gamma-proteobacteria] (two copies in one genome) from Low-G+C Firmicutes
*Vibrio* [Gamma-proteobacteria] (two copies in one genome) from Low-G+C Firmicutes
*Yersinia* [Gamma-proteobacteria] from Low-G+C Firmicutes
*Campylobacter* [Epsilon-proteobacteria] from Low-G+C Firmicutes
*Bacillus* [Low-G+C Firmicutes] (two species) from Proteobacteria
*Listeria* [Low-G+C Firmicutes] from Proteobacteria
*Oceanobacillus* [Low-G+C Firmicutes] from Proteobacteria
*Staphylococcus* [Low-G+C Firmicutes] (three strains in two species) from Proteobacteria
*Bifidobacterium* [High-G+C Firmicutes] from Proteobacteria
*Prochlorococcus* [Cyanobacteria] from Proteobacteria
*Fusobacterium* [Fusobacteria] from Proteobacteria
*Chlamydia* [Chlamydiae] from Proteobacteria
*Deinococcus* (two copies in one genome) from Proteobacteria

**LepA**
*Bifidobacterium* [High-G+C Firmicutes] from Thermus-Deinococcus
*Lactococcus* [Low-G+C Firmicutes] from Proteobacteria
*Deinococcus* from High-G+C Firmicutes

**Section 4.2 GO:0006412 translation: 964 genes in Dataset 2a (EB dataset)**

Ribosomal proteins = 583
Translation elongation factors = 40
Translation initiation factors = 40
Translation miscellaneous (probably non-core) = 6
tRNA synthetases / ligases = 130
Metabolic/operational & other non-core-translational = 165

See Section 4.1 (above) for an introduction to the "complexity hypothesis". Here we consider the over-enrichment of genes annotated with GO:0006412 in the EB dataset under three different groupings: 64 *E. coli* (EC) and *Shigella* (S) genomes combined into a single group (Dataset 2a), all ECS genomes removed, or only these S genomes removed. When these EC and S genomes are included but grouped separately, genes annotated with GO:0006412 translation become under-represented (see text).

The numbers below refer to TF-IDF inference on Dataset 2a (all ECS combined in a single group) at the default (mean value) IDF threshold. Of the 964 "translational" genes inferred as potentially lateral (Table S12), 130 encode tRNA synthetases/ligases, *i.e.* are well-known to be susceptible to LGT [1-3]. A further 165 encode metabolic/operational or other biological processes, and as such are not suspected of being LGT-resistant. Manual examination of a subset confirmed that the annotation is not in error, although the connection with translation can be indirect, *e.g.* as part of a specialised regulatory mechanism.

This leaves genes encoding 583 ribosomal proteins, 40 elongation factors, 40 initiation factors and six miscellaneous proteins.

Elongation factors have been discussed in Part 3.1 above. For the 40 EFs we identify as having accepted LGT, the breakdown is: EF-2 (6 instances), EF-G (22), EF-P and related (10), EF-Ts (1) and EF-Tu (5).

Jain *et al*. [4] included initiation factors 1, 2 and 3 among the translational apparatus components whose genes should be less-susceptible to LGT. For the 40 IFs we identify as having accepted LGT, the breakdown is: IF-1 (13), IF-2 (18) and IF-3 (9). We do not know of prior reports of LGT involving these genes.

Ribosomal proteins (r-proteins) comprise by far the greatest single component of this set. Collectively these sequences are considered to provide a conservative vertical central signal [4], and a subset of 16 r-proteins has been used to infer a three-Domain tree [5]. Individually, however, r-proteins are short, compositionally biased within and across Domains [4], and difficult to align [6]. Moreover, topologies of the inferred trees depend strongly on how the poorly alignable sites are treated [6]. For many although not all r-protein families, further complication is provided by multiple gene losses, restricted phyletic distributions and/or the presence of potentially subfunctionalised paralogs [7]. Yutin *et al*. [4] identified paralogs in 536 of

995 analysed bacterial genomes. All divergent paralogs contain a zinc-binding motif (zinc ribbon) [4,8], opening a path for (true or false) detection of LGT based on presence or absence of this conservative motif.

Keeping the above provisos in mind, LGT has previously been inferred for r-proteins S4 [8], S14 [9,10], S18 [7], L16 [10], L22 [10], L23 [6], L27 [11], L28 [7], L31 [7], L32 [7], L33 [7] and L36 [7]. Our list of r-proteins inferred by TF-IDF as affected by LGT includes most of these, and many others (S1 through S14, S16 through S21, L1 through L7, L9 through L11, L13 through L25, L25p, L27, L29, L30, L32 and L36).

Finally, in many bacteria and archaea, genes encoding many r-proteins occur adjacent to one another on the chromosome. Combined with the short length characteristic of r-protein genes, this means that an inferred lateral region that maps to the gene for one r-protein has an excellent chance of impinging on the gene for another. Given the overall weak gene-order conservation across and within many groups of Bacteria and Archaea, this "neighbour effect" is likely to affect r-proteins, hence the biological function *translation*, more than almost any other category.

**References for Section 4.2**


[1] Wolf, Y.I., Aravind, L., Grishin, N.V. & Koonin, E.V. Evolution of aminoacyl-tRNA synthetases – analysis of unique domain architectures and phylogenetic trees reveals a complex history of horizontal gene transfer events. *Genome Res.* **9**, 689-710 (1999).

[2] Woese, C.R., Olsen, G.J., Ibba, M. & Söll, D. Aminoacyl-tRNA synthetases, the genetic code, and the evolutionary process. *Microbiol. Mol. Biol. Rev*. **64**, 202-236 (2000).

[3] Kanhere, A. & Vingron, M. Horizontal gene transfers in prokaryotes show differential preferences for metabolic and translational genes. *BMC Evol. Biol*. **9**, Art. 9 (2009).

[4] Yutin, N., Puigbò, P., Koonin, E.V. & Wolf, Y.I. Phylogenomics of prokaryotic ribosomal proteins. *PLoS ONE* **7**, Art. e36972 (2012).

[5] Hug, L.A., *et al*. A new view of the tree of life. *Nat. Microbiol*. **1**, Art. 16048 (2016).

[6] Hansmann, S. & Martin, W. Phylogeny of 33 ribosomal and six other proteins encoded in an ancient gene cluster that is conserved across prokaryotic genomes: influence of excluding poorly alignable sites from analysis. *Int. J. Syst. Evol. Microbiol.* **50**, 1655-1663 (2000).

[7] Makarova, K.S., Ponomarev, V.A. & Koonin, E.V. Two C or not two C: recurrent disruption of Zn-ribbons, gene duplication, lineage-specific gene loss, and horizontal gene transfer in evolution of bacterial ribosomal proteins. *Genome Biol* **2**, Art. RESEARCH0033 (2001).

[8] Chen, K., Roberts, E. & Luthey-Schulten, Z. Horizontal gene transfer of zinc and non-zinc forms of bacterial ribosomal protein S4. *BMC Evol. Biol.* **9**, Art. 179 (2009).

[9] Brochier, C., Philippe, H. & Moreira, D. The evolutionary history of ribosomal protein RpS14: horizontal gene transfer at the heart of the ribosome. *Trends Genet.* **16**, 529-533 (2000).

[10] Coenye, T. & Vandamme, P. Organisation of the S10, spc and alpha ribosomal protein gene clusters in prokaryotic genomes. *FEMS Microbiol. Lett.* **242**, 117-126 (2005).

[11] Garcia-Vallvém *et al*. Simultaneous horizontal gene transfer of a gene coding for ribosomal protein L27 and operational genes in *Arthrobacter* sp. *J. Mol. Evol.* **55**, 632-637 (2002).

**Table S12. Details of gene types within GO:0006412 translation**

**Ribosomal proteins = 583**
Ribosomal proteins (small subunit) = 289
Ribosomal proteins (large subunit) = 294

**Metabolic/operational & other = 165**
ABC transporter ATP-binding protein = 2
Acetyl-CoA carboxylase subunit beta = 6
Aconitase hydrases & hydratases = 32
Aspartate-ammonia ligase (asparagine synthetase A) = 1
ATP-binding component of transport system (putative) = 2
ATT-dependent RNA helicase DeaD = 13
Carbamoyl-phosphate synthase large chain / large subunit = 35
Cold-shock DEAD-box protein A = 2
DEAD/DEAH box helicase domain protein = 8
GTP-binding protein = 7
GTP-binding protein TypA (includes TypA/BipA) = 9
HF-I host factor = 1
Host factor I for bacteriophage Q beta replication = 2
Hypothetical proteins = 26
Integration host factor beta subunit = 1
N5-glutamine SAM-dependent methyltransferase = 1
Peptide deformylase = 1
Ribosome recycling factor = 1
RNA chaperone /binding protein Hfq = 9
RNA helicases = 4
Short-chain dehydrogenase/reductase SDR = 1
Sigma modulation protein (putative) = 1

**Translation miscellaneous (probably non-core) = 6**
Energy-dependent translational throttle protein EttA = 1
GTP-binding elongation factor family protein = 2
Peptide chain release factor 3 = 1
Peptidyl-tRNA hydrolase = 1
Release factor (putative) = 1

**Translation elongation factors = 40**
Translation elongation factor 2 = 2
Translation elongation factor G = 22
Translation elongation factor P = 9
Translation elongation factor P – (R) beta lysine ligase = 1
Translation elongation factor Ts = 1
Translation elongation factor Tu = 5

**Translation initiation factors = 40**
Translation initiation factor 1 = 13

Translation initiation factor 2 = 18
Translation initiation factor 3 = 9

**tRNA synthetases / ligases = 130**
Asparaginyl = 23
Glutaminyl = 9
Glycyl = 3
Isoleucyl = 1
Lysyl = 5
Methionyl = 4
Prolyl = 14
Threonyl = 2
Valyl = 69
